# The fundamentals of eye tracking part 6: Working with areas of interest

**DOI:** 10.3758/s13428-025-02937-3

**Published:** 2026-02-17

**Authors:** Ignace T. C. Hooge, Marcus Nyström, Diederick C. Niehorster, Richard Andersson, Tom Foulsham, Antje Nuthmann, Roy S. Hessels

**Affiliations:** 1 Experimental Psychology, Helmholtz Institute, Utrecht University, Utrecht, The Netherlands; 2 Lund University Humanities Lab, Lund University, Lund, Sweden; 3 Department of Psychology, Lund University, Lund, Sweden; 4 Tobii AB, Danderyd, Sweden; 5 Department of Psychology, University of Essex, Colchester, UK; 6 Institute of Psychology, Kiel University, Kiel, Germany

**Keywords:** Eye tracking, Area of interest, Operationalization

## Abstract

Researchers use area of interest (AOI) analyses to interpret eye-tracking data. This article addresses four key aspects of AOI use: 1) how to report AOIs to support replicable analyses, 2) how to interpret AOI-related statistics, 3) methods for generating both static and dynamic AOIs, and 4) recent developments and future directions in AOI use. The article underscores the importance of aligning AOI design with the study’s conceptual and methodological foundations. It argues that critical decisions, such as the size, shape, and placement of AOIs, should be made early in the experimental design process and should involve eye-tracking data quality, the research question, participant tasks, and the nature of the visual stimulus. It also evaluates recent advances in AOI automation, outlining both their benefits and limitations. The article’s main message is that researchers should plan AOIs carefully and explain their choices openly so others can replicate the work.

## Introduction

This article is the sixth installment in a series on the fundamentals of eye tracking. The articles are aimed at individuals who are (one of) the first in their group, company, or research field to use eye tracking, with a focus on all the decisions one may make in the context of an eye-tracking study. Such individuals may come from academia (e.g., psychology, biology, medicine, educational science, computer science), commercial institutions (e.g., marketing research, usability, decision making) and non-commercial institutions (e.g., hospitals, air traffic control, military organizations). Note that this is not an exhaustive description of the target audience. More experienced eye-tracking researchers may find useful insights in the article series, or may find the article series a useful reference or hub to relevant research. Previous papers in the series have dealt with (1) the relation between theory and research question (Hessels et al., 2025c), (2) operationalizing research questions (Hooge et al., 2025b), (3) choosing an eye tracker (Nyström et al., 2025), (4) tools for eye tracking studies (Niehorster et al., 2025b), and (5) the importance of piloting (Hessels, Niehorster, Nyström, Andersson, Holleman, & Hooge, 2025b). One may choose to start by reading the present article, but one may also first read earlier articles in the series. The present article addresses *working with areas of interest*.

### The definition of the term AOI

According to Holmqvist et al. (2011, p. 187), an area of interest is a region within the visual stimulus that the researcher intends to examine for the corresponding eye-tracking data. In the literature, AOI is not the only term referring to such an area. We also find region of interest (e.g., Akahori, Hirai, Kawamura, & Morishima, 2016; Al-Azawi, Yang, & Istance, 2014; Hofbauer, Kuhn, Püttner, Petrovic, & Steinbach, 2020), interest region (e.g., Dupont, Antrop, & Van Eetvelde, 2014) and interest area (SR Research Data Viewer software). In this article, we will use only the term AOI.

### The motivation for this article

You might wonder why we chose to write an article on AOIs, given that there is already an excellent book chapter dedicated to this topic (Holmqvist et al., 2011, Chapter 6). That chapter offers a wealth of information, including descriptions of various types of AOIs and associated AOI measures. In our view, at least two important aspects are somewhat underemphasized in Holmqvist et al. (2011): 1) the methods used for AOI production, and 2) what an AOI signifies in terms of, for instance, perception, attention, and visual information processing. Furthermore, the field has evolved considerably since 2011. Most notably, wearable eye tracking has matured significantly, computing power has increased and numerous new algorithms have emerged that support automated AOI generation and annotation (e.g., Alinaghi, Hollendonner, & Giannopoulos, 2024; Batliner, Hess, Ehrlich-Adám, Lohmeyer, & Meboldt, 2020; Deane, Toth, & Yeo, 2023; Duchowski, Gehrer, Schönenberg, & Krejtz, 2019; Gehrer, Svaldi, & Duchowski, 2024; Hessels, Benjamins, Cornelissen, & Hooge, 2018a; Hessels et al., 2020; Kopácsi, Barz, Bhatti, & Sonntag, 2023; Tzamaras, Wu, Moore, & Miller, 2023; Wolf, Hess, Bachmann, Lohmeyer, & Meboldt, 2018). We advise researchers new in the field to read both (Holmqvist et al., 2011) and this article together to get a good overview.

### The scope of this article

AOIs have been used in eye movement research since the 1970s. However, this concept is used in at least two ways in the eye movement literature. Holmqvist et al.’s definition primarily reflects the AOI as a *top-down* research tool. A researcher might create several AOIs and, after conducting an eye tracking study, compare AOI statistics (e.g., time spent in the AOI, time to first AOI entry, number of fixations within the AOI). Here, the AOI serves as an analysis tool to study human gaze behavior. The other use appears to be more of a *bottom-up* description of empirical gaze data. By *bottom-up* we refer to the approach of identifying regions that receive a high number of fixations based on the data. For example, Janik, Wellens, Goldberg, and Dell’Osso (1978, p. 858) write: “The results, while certainly not conclusive for all studies dealing with looking at others, add support to Argyle’s (1970) notion that the eye region represents a prime area of visual interest”. Sakano (1963) describes that, depending on the instruction, the facial or mouth areas attract more or fewer looks. The difference between the two uses of AOI is subtle but significant. In this article, we mainly focus on the a priori definition of interesting regions before looking at the data.

This article is not an update of Chapter 6 from Holmqvist et al. (2011). That chapter discusses types of AOIs (e.g., gridded, dynamic, and distributed AOIs), AOI statistics, and analyses based on those statistics (e.g., entropy, scan paths). In contrast, the current article focuses on: how to report on AOIs to support replicable analyses, how to interpret AOI statistics, methods for producing AOIs, and future directions of AOI use. This article is not a guideline. It is intended to encourage authors to think more deeply about the many decisions involved in AOI design and production. It is also meant to make clear that AOI production is more than a practical and technical problem; it also involves philosophical and methodological considerations.

**Fig. 1:**
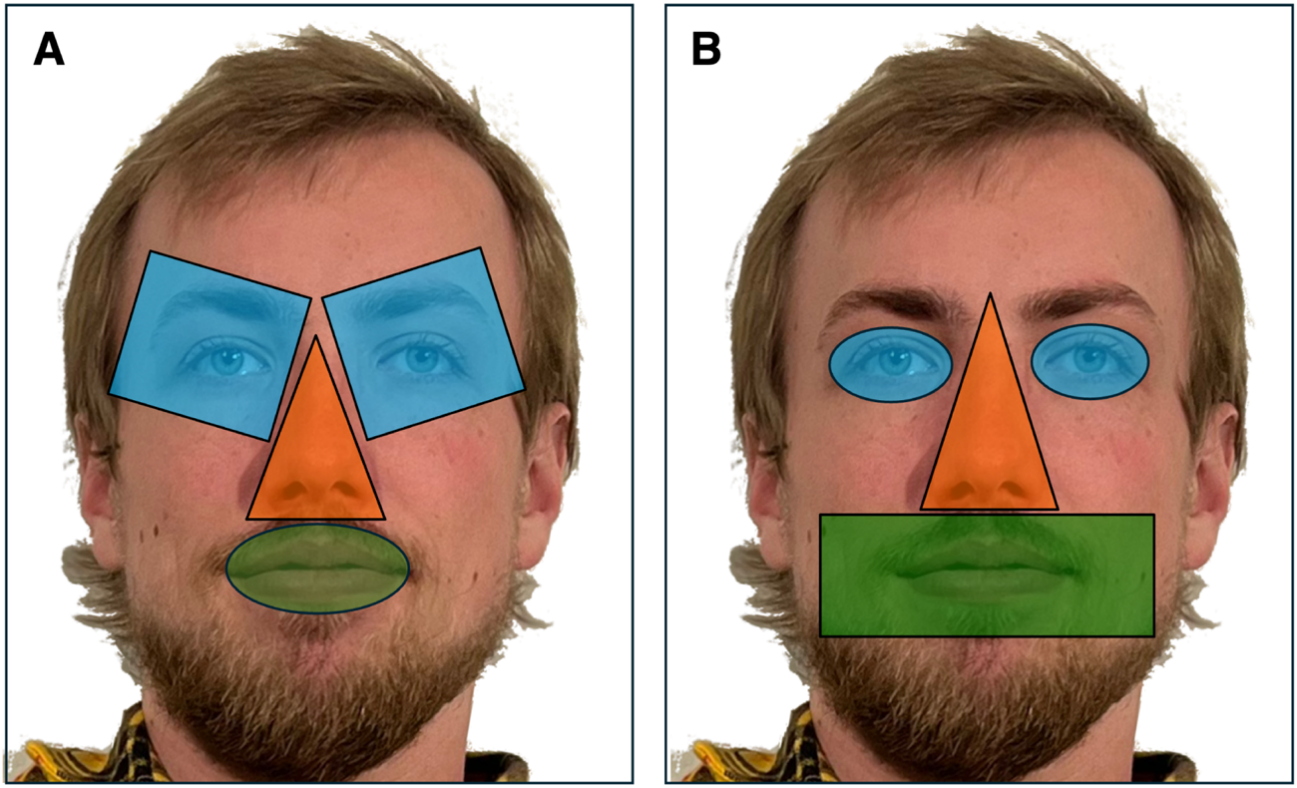
Two hand-drawn AOI sets. (**A**) and (**B**) show subjective hand-drawn eye, nose, and mouth AOIs. An AOI analysis may produce different outcomes depending on the set used because of the different shapes, positions, sizes, and orientations of the AOIs. By means of this (overstated and rather unrealistic) example, we emphasize that accurate description (e.g., size, shape, orientation, and position) of the AOI is essential in comparing and understanding different study outcomes. The description of the AOI can be in the form of illustrations, polygon coordinates, or scripts showing how to automatically produce them

## Reporting about AOIs

A crucial aspect of working with AOIs is the accurate reporting of the AOIs used in the methods section. In our view, current reporting practices are insufficient. With this article, we aim to encourage more thorough and transparent documentation of AOIs. In the following section on the “The hypothesis and the AOI”, we explain how AOIs relate to experimental hypotheses and argue that this relationship may be more flexible than often assumed. We then explore, using studies with different AOIs, whether AOIs can be reproduced. It should be clear that reproducing AOIs is essential for replicating a study. Through this discussion, we aim to provide readers with practical guidance on how to report their own AOIs in future publications.

### The hypothesis and the AOI

Holmqvist et al. (2011, p. 188) write that AOIs are part of a study’s hypothesis, and they argue that if a researcher manipulates the AOI size, shape, or position, the researcher implicitly alters the hypothesis. We disagree with this statement, but agree that the hypothesis (expressed as language) may have a strong relation with the AOIs (parts of images^1^). We consider AOIs to be an operationalization (Hooge et al., 2025b) of the hypothesis, not a part of the hypothesis itself. If two identical studies have operationalized the hypothesis differently, the conclusions of the study may be affected by this. Let us illustrate this with a hypothetical eye-tracking study conducted in two different labs (one in Utrecht and one in Lund) on how faces are viewed, specifically focusing on gazing at the eyes or the mouth. Figure 1 shows two sets of AOIs for the eyes, nose, and mouth for the same face stimulus (the left set for the lab in Utrecht, the right set for Lund). If the Utrecht and Lund labs test the same hypothesis, these studies may report different outcomes based on the differences between the AOI sets. This exaggerated example highlights the importance of 1) careful production of AOIs, and 2) careful reporting on the spatial properties of the AOIs and/or how the AOIs were created, as this is critical for replicating and comparing studies.^2^ How do researchers describe their AOIs in their method sections, and can their AOI analyses be reliably repeated based on this information?

**Fig. 2:**
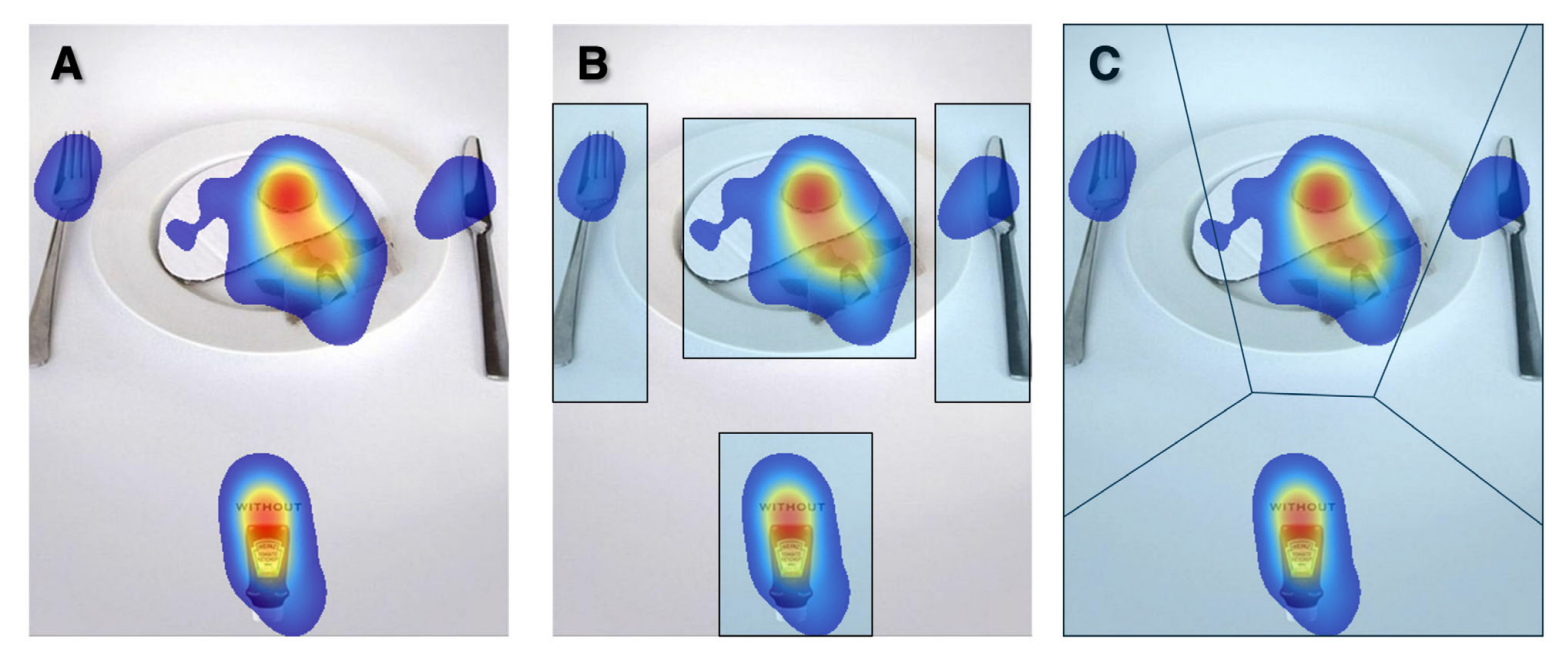
Example of a sparse display with AOIs. (**A**) A sparse visual display with a heatmap plotted on top. The heat map represents fixations from about 40 participants. *Hotter colors* indicate higher fixation density. Fixation time was not taken into account in the heat map visualization. (**B**) Small hand-drawn AOIs with surrounding white space. (**C**) Large AOIs adjacent to each other produced by the Voronoi method (see Fig. 1, Over, Hooge, & Erkelens, 2006). (**B**) and (**C**) show that in sparse stimulus displays, for the AOI statistics, the size of the AOI (as long as the AOI is above a certain minimum size) does not matter much for the AOI statistics, because fixations are primarily focused on one of the four objects present (fork, knife, ketchup bottle, and plate with egg). Evaluating heat maps may help researchers develop an intuition for designing AOIs

### AOI reproduction

Imagine that a researcher wants to replicate a study and as a part of this repeat the AOI analysis. How easy is it to copy an AOI set from another study? And how similar do these AOIs need to be, to be able to replicate the published study? The answer to this question may depend on the number of AOIs, the stimulus displays, the eye tracking data quality, the task instruction for the participants, the AOI production method, the type of replication (Hudson, 2023) and how the study reports all these things.

The simplest AOIs to reproduce are those that divide an entire screen into, for example, two, four, nine or more sections or include only a few large AOIs. Examples of such studies include (Foulsham & Underwood, 2008; Foulsham, Wybrow, & Cohn, 2016; Glaholt, Wu, & Reingold, 2009; Schotter, Berry, McKenzie, & Rayner, 2010; Shimojo, Simion, Shimojo, & Scheier, 2003; van der Laan, Hooge, de Ridder, Viergever, & Smeets, 2015).

AOIs may be easier to reproduce in displays that are more sparse. Sparse displays would include those with only a few objects, which are easily distinguishable from the background, or where the majority of the display is empty.

Examples of sparse displays include displays with a single face (Fig. 1) and the ketchup advertisement from Fig. 2. On the other hand, clear examples of dense displays include the panda search display (Fig. 4), the scene image used in Fig. 7 and panel A of Fig. 9.

In sparse displays, exact replication of the large AOIs is less critical, because most fixations are on the objects rather than between them (Nuthmann, De Groot, Huettig, & Olivers, 2019) and since the objects are far apart compared to sizes of the objects, it is clear which fixations belong to which object. In naturalistic scenes, which tend to be more cluttered, observers often fixate near the center of objects, and the distributions of these fixations typically resemble a two-dimensional Gaussian (Nuthmann & Henderson, 2010). For smaller objects, however, the distributions may be truncated, suggesting that not all fixations land on the intended object (Pajak & Nuthmann, 2013). The object center-bias has been observed using a variety of AOI definitions, including rectangular AOIs with a margin (Nuthmann & Henderson, 2010), without a margin (Foulsham & Kingstone, 2013; Nuthmann, Schütz, & Einhäuser, 2020), and closely fitting polygonal AOIs (Borji & Tanner, 2015). Still, the choice of AOI may affect the proportion of fixations assigned to the object because, e.g., rectangular AOIs cover more area than polygonal ones, and margins further increase coverage.

Other classes of easy-to-copy AOIs are found in eye tracking studies:

- Using computer-generated (search) displays (e.g., Gilchrist & Harvey, 2000; Hessels, Hooge, & Kemner, 2016a; Körner & Gilchrist, 2008; Najemnik & Geisler, 2005; Trukenbrod & Engbert, 2007). Because the researchers have programmed the stimulus displays themselves, the nature and location of each stimulus element are known (e.g., in a log file produced by the stimulus generation program). Rutishauser and Koch (2007) write: “The trial automatically terminated as soon as the subject fixated for at least 320 ms in a radius of 1.5-around the target”. This is an example where the AOI is not explicitly mentioned.
- With only one (target) AOI per trial (e.g., Nuthmann & Clark, 2023). If the number of trials is not too large, a list with the coordinates of the target AOIs can be manually created. An AOI can be defined as an area around the target center. Replication requires that the stimulus material is available and/or that the target AOIs are well described.
- Using virtual reality environments. The advantage of VR is that for all objects in the scene at any given time locations, and orientations are known. Clay, König, and Koenig (2019, p. 2) write: “Contrary to real-world eye tracking, it is easy in VR eye tracking to define regions of interest in 3D space and trace the points in time to determine when the regions were looked at”. However, reproducing AOIs from a VR study is only easy if the study shares code or information about objects and AOIs.
- With automatically generated AOIs on words in texts (Tang, Reilly, & Vorstius, 2012), on elements in websites (Eraslan, Yesilada, & Harper, 2020) and on salient regions in static displays (Fuhl, Kübler, Santini, & Kasneci, 2018).
- With computer-vision methods for AOI production. For instance, OpenFace (Baltrušaitis, Robinson, & Morency, 2016) is used to generate facial markers for defining face AOIs (Duchowski et al., 2019; Hessels et al., 2018a; Vehlen, Standard, & Domes, 2022). Similarly, OpenPose (Osokin, 2018) is employed to extract skeleton joint positions for creating body AOIs (Gehrer et al., 2024; Hessels et al., 2020; Müller & Mann, 2021).
- Used to monitor whether participants look at the central fixation marker when they are not allowed to make saccades to peripheral targets (Christ & Abrams, 2006; Galfano et al., 2012; Patching & Jordan, 1998). There is often only one AOI (in the center of the screen).

One of the problems with reproducing AOIs from most of the eye-tracking studies is that these studies do not report, or report too briefly, about the shape and size of the AOIs and how they were produced. An example is the study by Amati, Parmehr, McCarthy, and Sita (2018). It is not clear how the AOIs were created—probably with software, as this concerns dynamic AOIs. The first author of the current article has also been guilty of not clarifying how it was determined whether participants located the target in a visual search task, or which elements they fixated on during the search (Hooge & Erkelens, 1999). Gilchrist and Harvey (2000, p. 1212) did a better but not perfect job in describing the analysis without using the term AOI. They wrote:

The stimulus display consisted of 31 capital letters arranged randomly at 31 of the 49 intersections of an imaginary 7 by 7 grid, with blank spaces appearing in the other 18 locations. There was a $2^{\circ }$ center-to-center spacing between the intersections. Fixations classified as being a repeat fixation on the same location were combined together and for all the analyses reported counted as a single fixation. Fixations outside the display area, fixations to blank locations between item boxes and fixations on the target at the end of the search were all excluded from the analysis.

In general, articles should include at least basic information about the nature of the AOIs (e.g., circles, free forms, rectangles), how they were defined, whether any software was used (and if so, which one), and, if possible, whether the actual visual stimuli and AOIs can be shared.

Repeating an AOI analysis might become more challenging if the original study involves hand-drawn AOIs with real-world stimulus material, such as faces (e.g., Falck-Ytter, 2008; Jones & Klin, 2013), landscapes (Zhou et al., 2022) or newspapers (Wartenberg & Holmqvist, 2005). However, some authors provide readers with their stimulus material and well-documented AOIs (e.g., Jiang, Chen, & Kang, 2021; Xu, Jiang, Wang, Kankanhalli, & Zhao, 2014). The sections below on “Static AOIs” and “Dynamic AOIs” sections discuss methods of AOI production.

## The meaning of an AOI hit, the size of an AOI and perception of the peripheral field

With an eye tracker, one can directly determine where an observer looked, but not what they actually saw. For example, visual search studies have shown that observers sometimes fixate the target but then continue searching before eventually returning to it (e.g., Clayden, Fisher, & Nuthmann, 2020; Hooge & Erkelens, 1996; Rutishauser & Koch, 2007). This raises the question of what exactly a fixation within an AOI indicates? Does it imply that the observer has consciously noticed the content within the AOI, that the content is influencing oculomotor control, that the observer is attending to it, or that the brain has processed it? Unless explicitly tested with an experiment, there is no definitive answer – each interpretation may depend on context and assumptions.

But what if the observer did not fixate within an AOI? In some cases, the implications are clearer. For instance, if an observer views an outdoor advertisement but never looks near the brand logo – such that it’s too far from the point of fixation to be perceivable – then one can confidently say that the brand logo was not consciously noticed, attended to, or processed. At the very least, it may be assumed that the likelihood of the content of an AOI being consciously noticed, involved in oculomotor control, attended to, or processed by the brain is higher when the AOI is fixated, compared to when it is not. This view differs from the strict interpretation of the eye-mind hypothesis (Just & Carpenter, 1976), which asserts that anything looked at has, in some form, been cognitively processed. In this context, we would like to highlight the more nuanced discussions of the eye-mind hypothesis in Hessels et al. (2025c); Hooge et al. (2025b); Viviani (1990). Foulsham and Kingstone (2025) argue that, particularly in realistic social settings, elements outside the direct line of gaze are being selected and processed. The extent to which elements located far from the fixation point are being processed or perceived depends on factors such as object size, retinal resolution, crowding, and the task at hand (see also Rosenholtz, 2024).

Does the uncertainty of whether an AOI has been cognitively processed make eye tracking seem less appealing as a research tool? We believe it does not. However, it does make conducting eye tracking research more complex. Saccades and fixations can be used effectively to study a wide range of topics. However, potentially interesting concepts such as attention, perception, message transfer, and learning – cannot be measured directly. What we can do is approach them by using eye movements as a proxy (Hooge et al., 2025b; Hessels et al., 2025c). In this proxy approach, the AOI and the AOI analysis play an important role.

**Fig. 3:**
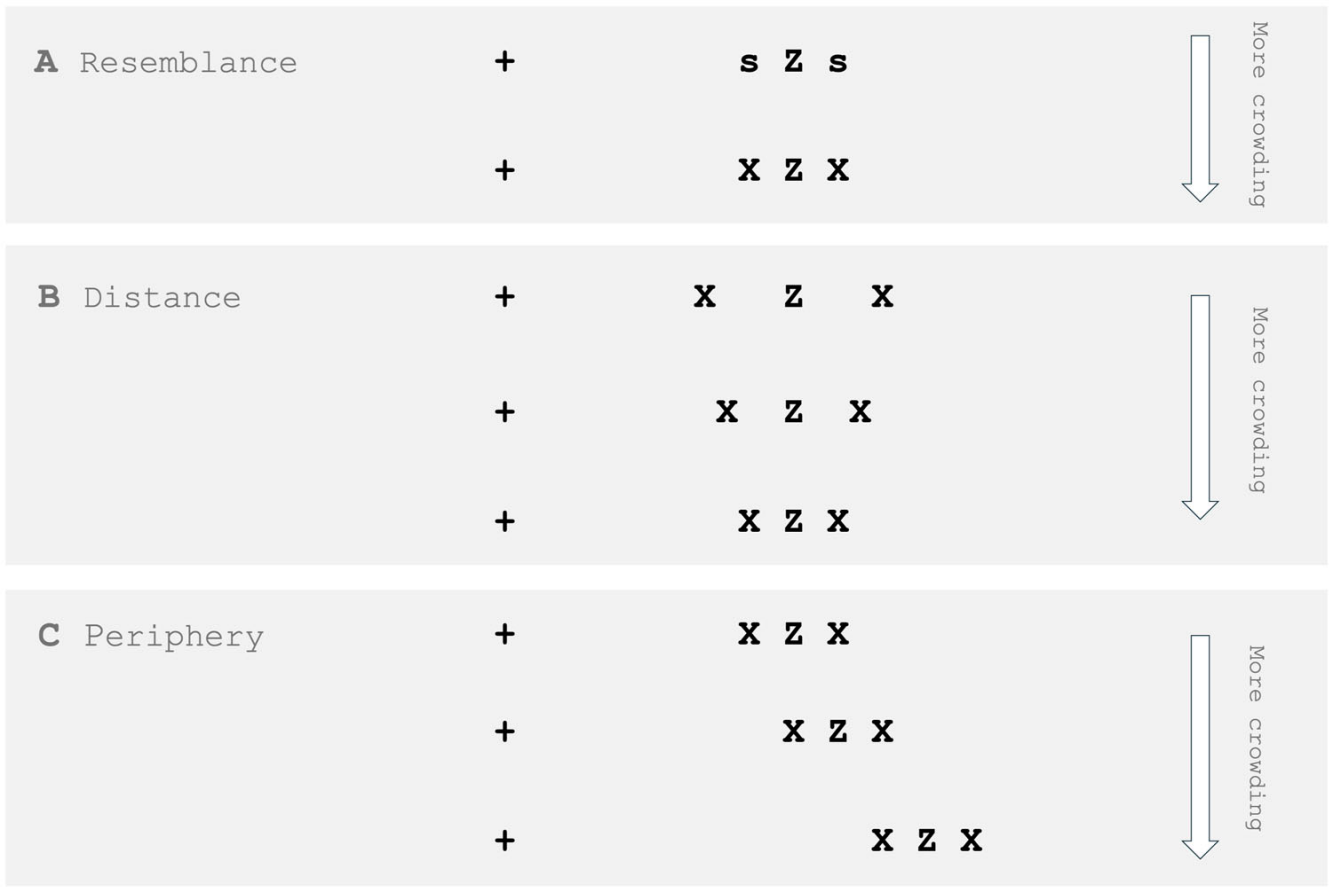
How far away from the target can one fixate and still identify it? (**A**) If one looks at the fixation marker (+), the target (Z) becomes more crowded (less conspicuous) if the flankers resemble the target more. (**B**) The target becomes more crowded if the flankers are closer to the target. (**C**) The target becomes more crowded if the flankers and the target are further in the periphery. In general, in sparse visual displays, perception is less hampered by crowding, indicating that one can look further away from an object and still identify it

### Perception of an object while fixating next to it

An important decision when creating AOIs is the size of the AOI. Do we draw the edges of an AOI tightly around the object, or do we make the AOI a bit larger by adding a margin? Before we can discuss the optimal or desired size of an AOI, it’s important to first consider the role of visual perception. An observer may not need to look directly at the center of an object to be able to identify it. A key question is: how far away from an object can an observer look and still recognize it? Eye tracking can’t answer this question. To determine what an observer can actually identify, psychophysical research is needed. A substantial body of research has explored how far one can divert one’s gaze from an object and still perceive it. Initially, it was thought that retinal resolution was the only bottleneck for object recognition in the periphery, given that acuity degrades with increasing distance from the fixation location (Rosenholtz, 2016). For peripheral identification in sparse stimuli, retinal resolution may indeed be the bottleneck: an object can be too small to be identified by the information gathered by the peripheral retina. Following research into a phenomenon known as *crowding* (e.g., Bouma, 1970; Kooi, Toet, Tripathy, & Levi,^1994^; Levi, 2008; Pelli & Tillman, 2008; Toet & Levi, 1992; Vlaskamp & Hooge, 2006; Whitney & Levi, 2011), it has become clear that the visual content of the immediate surroundings of a target influences how far from a target one can look and still identify or see it. Pelli and Tillman (2008) describes crowding as a failure of object recognition that occurs in the presence of visual clutter. In particular, they note that objects which are easily identified on their own become difficult or even impossible to recognize when presented alongside surrounding stimuli. Knowledge of crowding may help make decisions about how big or small to draw the AOIs. If the local stimulus allows observers to perceive objects only when fixating them, the edges of an AOI could be drawn tightly around the object.^3^ The AOI can be enlarged by adding a margin, or substantially increased in size (as in Fig. 2C) when observers are able to identify the object without directly fixating on it. How big or small one could make an AOI will be discussed in the “Should the AOIs be small or large?” section. For a more elaborate explanation about crowding and peripheral vision, we point the readers to review articles on crowding by Pelli and Tillman (2008) and Levi (2008) and about peripheral vision by Rosenholtz (2016).

The stronger the crowding, the less conspicuous the target, and the smaller the distance between the fixation point and the target required to identify it. Figure 3 illustrates the three key rules of crowding (Bouma, 1970; Kooi et al., 1994; Toet & Levi, 1992). The first rule reads: if a target physically resembles its local neighboring elements^4^ more closely (e.g., shape, size, color, orientation), the crowding effect is stronger than when the target is less similar to its neighboring elements (Fig. 3A). The second rule is that if the neighboring elements are placed closer to the target, the crowding effect becomes stronger (Fig. 3B). The first two rules of crowding are used in camouflage as a strategy in military contexts. If a soldier does not want to be noticed among bushes, they should resemble a bush as much as possible in terms of local contrast, shape, color, and size, and then position themselves close to the real bushes. The third rule states that if the target and its neighboring elements are located further in the periphery, the crowding effect becomes stronger (Fig. 3C). In summary, in the case of strong crowding, a target stands out less from its background and it can only be identified if the fixation point is close enough to the target.

**Fig. 4:**
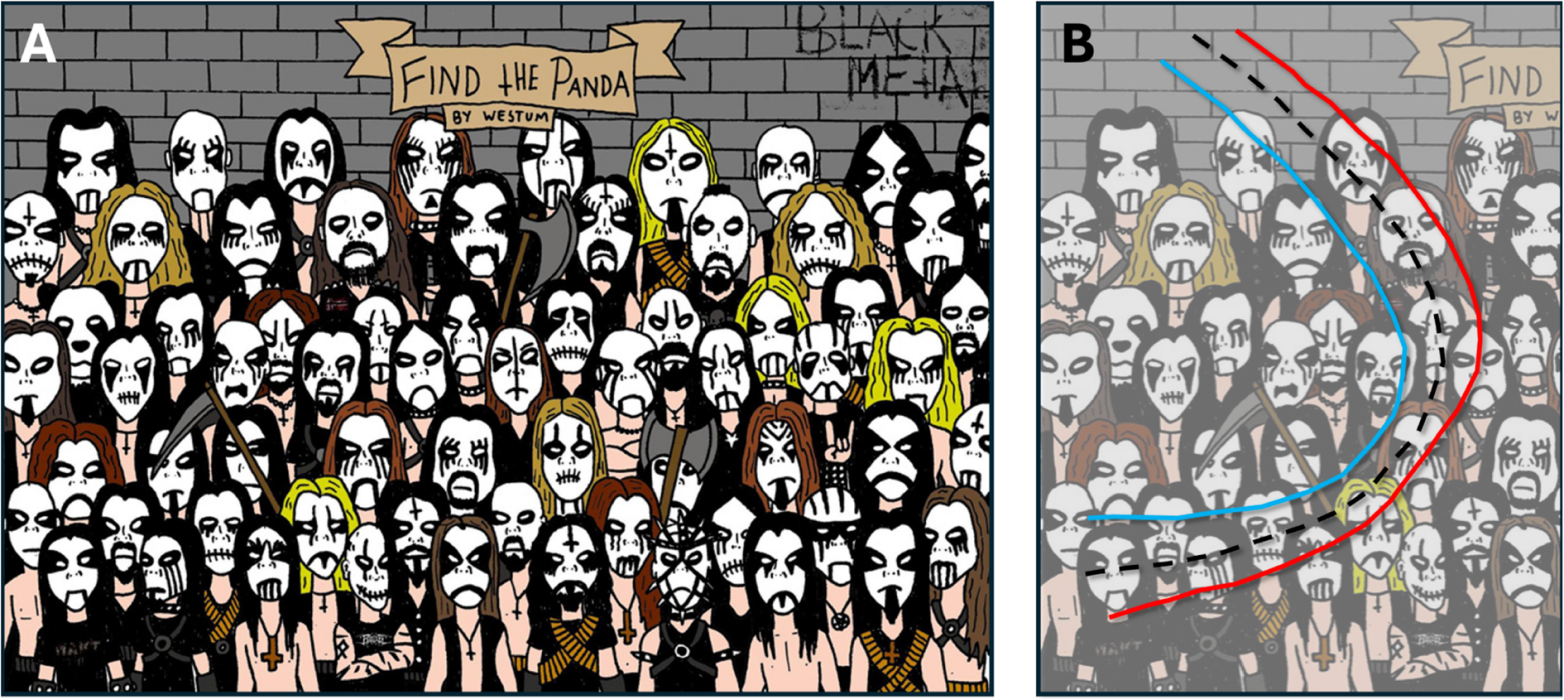
The conspicuity area and the naked eye method. (**A**) Among these black metal musicians, a panda is hidden. If you cannot find the panda, he is located on the third row on the left. (**B**) Results of the naked eye test carried out with a pen on a piece of paper (size A4). An experimenter moves a pen tip away from the panda and an observers says stop if he cannot identify the panda anymore. This is repeated a few times in different directions. The *red line* connects the points where the observer said stop. The previous procedure was repeated but now in the opposite direction (from far to close). The *blue line* connects the points where the observer said stop moving toward the panda. The *black dashed line* is based on the blue and the red lines and is a rough and quick estimate of the conspicuity area that enables object detection within one glance (Engel, 1977)

### The naked eye method

Before deciding about AOI size, we advise the reader to gain some intuition in how far an observer can look away from an object and still identify it. Engel (1977) termed the area that enables object detection within one glance the *conspicuity area* for that object. To estimate the size of the conspicuity area of a certain target, one could apply *The naked eye method* (Wertheim, 2010). Let us explain this rule with the example image in Fig. 4, which contains a search image from the popular *Find the panda* series (Westum, 2024). The experimenter uses a pen capped with its lid to point to the panda in the image. They then slowly move the pen away from the panda while the observer tracks the pen cap with their eyes. The observer says stop when the panda is no longer visible (identifiable). At that moment, the experimenter marks the corresponding point on the image. This procedure can be repeated in multiple directions to gather more data. The second step is repeating the previous in the opposite direction (starting far away from the panda). The experimenter places the capped pen further away from the panda than the previously measured distance and moves the pen toward the panda. The experimenter marks a point when the observer reports seeing the panda. This procedure could also be repeated in a few directions. Then the distances from the points (belonging to moving the pen away and toward the panda), could be averaged to get an estimate of how far the observer can look past the panda and either see or not see it (see the black dashed line in Fig. 4B). This method is based on the work of Bouma (1970) and Engel (1971) on the conspicuity area of a visual target. We believe that applying and experimenting with the naked eye method is a method for researchers to learn about using conspicuity areas to decide about AOI size and to substantiate AOI size with empirical data.

**Fig. 5:**
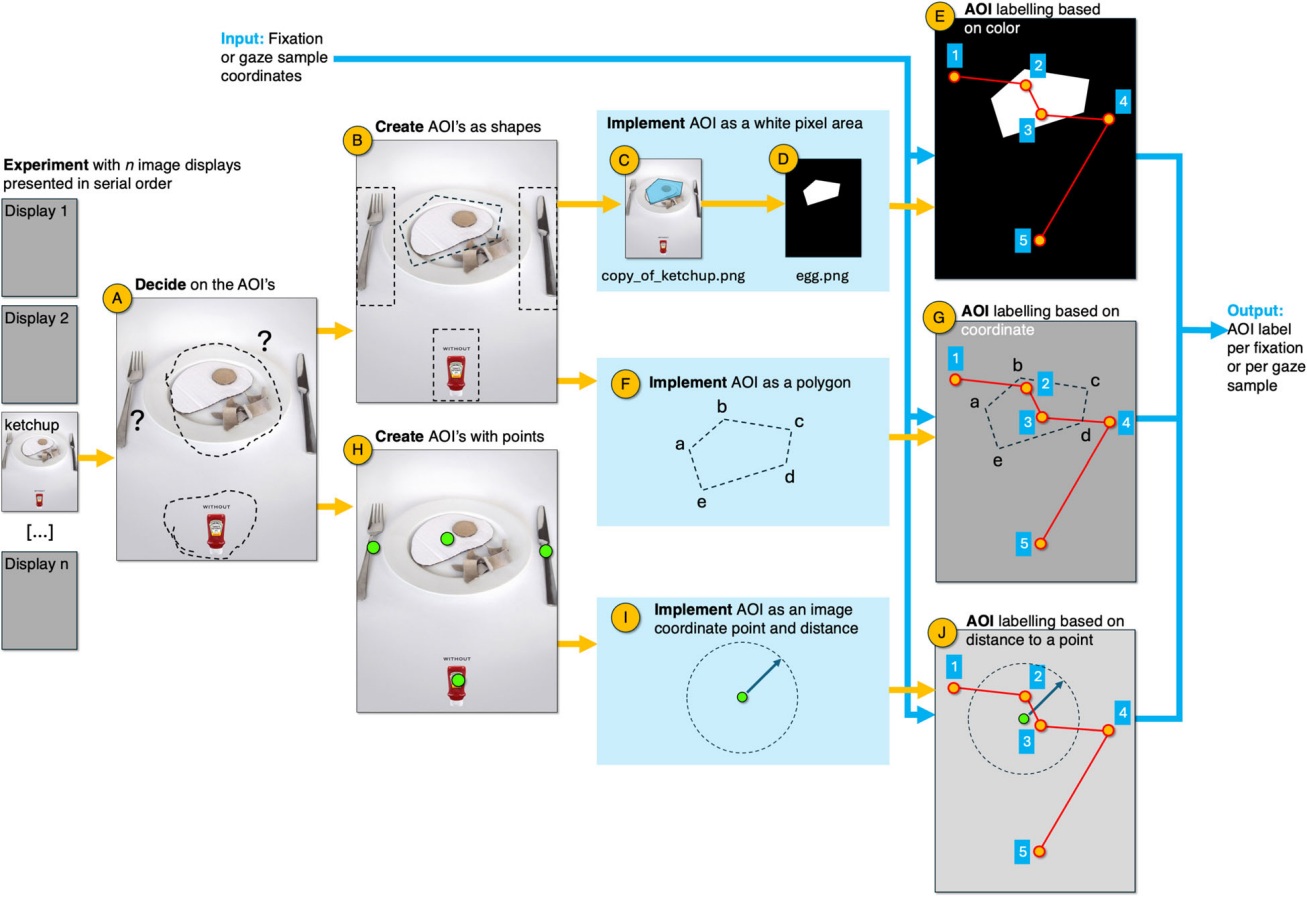
AOI production: from stimulus display to AOI label. (**A**) Conceptual foundations should guide AOI design. Key design decisions regarding AOIs are often made implicitly or too late in the research process. Ideally, these choices should be guided by careful consideration of several foundational methodological questions such as: Where are differences expected based on the experimental manipulations? What is the anticipated eye-tracking data quality? Will the analysis compare fixation durations, binary indicators of whether an item was viewed, or the sequence of fixations? Could it be a statistical issue if an AOI contains no data in at least one trial? AOI production should follow from this conceptual groundwork, ideally involving a focused discussion on the number, size, and shape of AOIs, the necessity of margins, and whether AOIs should be adjacent or spatially separated. (**B**) AOIs can be created based on shapes or (H) points (Hessels, Kemner, van den Boomen, & Hooge, 2016b; Rim, Choe, Scrivner, & Berman, 2021). The choice of the shapes or location of the points is usually subjective. (**C/D**) Based on shape, AOIs can be implemented as black-and-white (Boolean) pixel images (one image per AOI, same dimensions as the original display). (**E**) AOI labeling of the fixation or (gaze sample) can be performed based on the spatial coordinates, by checking the color of the pixel being looked at. If the color is white, the label becomes the name of the AOI. (**F**) Implementation of an AOI using a polygon description. (**G**) AOI labeling of a fixation (or gaze sample) based on the image coordinate (inside or outside the polygon). (**H**) AOIs can be defined by a point. (**I**) A possible implementation of the AOI is the distance between the AOI center (the point) and a fixation or sample coordinate (Hessels et al., 2016b). (**J**) In this case, labeling could be performed by choosing the nearest AOI point or by setting a maximum distance to an AOI point. The input of the labeling process is the spatial coordinate from the fixation of the gaze sample. The output of the labeling process is an AOI label (e.g., egg)

## Static AOIs

This section discusses how researchers can produce their own AOIs for studying eye movements in static displays (e.g., photos, X-rays, advertisements, paintings, magazine pages, and maps). The process of AOI production can be divided into three phases, namely, deciding on the AOI (Fig. 5A), creation of the AOI (Fig. 5B and H), and implementation of the AOI (Fig. 5C, D, F, and I). The different phases will be described below.

### Deciding on the AOI

In the context of human–computer interaction, according to Steichen (2024): “The definition of AOIs is typically performed manually, i.e., a designer/analyst explicitly defines the bounding coordinates for each interface-dependent AOI”. However, before one can draw or define individual AOIs, many decisions have to be made. One has to decide the number of AOIs, the size and shape of the AOIs, whether they should be contiguous or if space between the AOIs is allowed, and more. These decisions are worth a thorough discussion and many of the decisions on AOIs are related to the research question, the hypotheses and properties of the visual stimulus material. For example, when designing a visual stimulus, one might already account for the distance between objects to facilitate AOI analysis and more easily determine whether observers are looking at object A or object B. This approach has been used in reading research, where single lines of text are used, or lines are vertically spaced apart widely, so that each fixation can be easily assigned to a line.

In the literature, we see a variety of differently shaped and sized AOIs. For example, rectangular AOIs that do not touch each other (Drusch, Bastien, & Paris, 2014, Fig. 2), AOIs drawn precisely around an object (Russell, Torralba, Murphy, & Freeman, 2008), AOIs that cover almost an entire visual stimulus and fit closely together (Fuhl et al., 2018, Fig. 1c), overlapping AOIs (Meyer, Josefsson, Vrotsou, Westin, & Lundberg, 2021, Fig. 1), AOIs with a margin and without whitespace between them (Wenzlaff, Briken, & Dekker, 2018, Fig. 2), distributed AOIs (Pieters & Wedel, 2004, brand, pictorial, and text elements), very large AOIs with a significant margin around the object (Hessels et al., 2016b, Fig. 3 Voronoi), Gridded AOIs (Nuthmann et al., 2020),^5^ or the special case where a complete annotation is required, such as in text reading or certain scene perception studies, e.g., Figure 5 in Malcolm, Nuthmann, and Schyns (2014) or Figure 1 in Hayes and Henderson (2021). A question is how much it matters for the AOI statistics if the AOIs are contiguous, fit precisely around objects, have a margin, or are even overlapping. This is an empirical question, and pilot experiments may help in the decision-making about the AOIs (Hessels et al., 2025b). What also strikes us is that few authors provide a clear rationale for their choice of a particular type of AOI.

#### The empirical approach and piloting

Choices regarding the AOIs (e.g., size, spacing between AOIs, shape) may influence the AOI statistics (e.g., time spent in the AOI, time to first AOI entry, number of fixations in the AOI). Investigating how AOI statistics depend on choices related to the AOIs is, in our view, important for researchers. What researchers do not want is for the outcome of their study to hinge on minor variations in, for example, AOI size. In some cases, researchers may need to redo the AOI creation and analysis if reviewers question how much the study’s conclusions – such as whether individuals with diagnosis X look more at the mouth than the eyes compared to controls – depend on the size or shape of the eye and mouth AOIs. This can be treated as an empirical question, and it may be useful to conduct a sensitivity analysis by rerunning the analyses with slightly different AOIs. Clearly, researchers using automatically generated AOIs and programmed AOI analysis pipelines are at an advantage here because they can more easily manipulate AOIs and produce statistics. An example of such an analysis can be found in Fig. 6 of Hessels et al. (2016b), which showed that metrics like time to first AOI hit and dwell time (time spent in an AOI), remained similar across a wide range of AOI radii. We recommend the same empirical approach regarding the question of whether AOIs should be separated by space or touch each other. Researchers should test this using their specific stimuli and eye-tracking data. In general, to develop some intuition, researchers could run pilot AOI analyses on eye-tracking data from an existing study (Hessels et al., 2025b).

#### Should every object be assigned an AOI?

Let’s start with the question of whether every object in the stimulus display should get its own AOI (as in Fuhl et al., 2018, Fig. 1e and b). In many studies, that’s not necessary at all. The number of AOIs required is usually related to the research question. A good example is the marketing study by Pieters and Wedel (2004). They were interested in attention to the three key advertisement elements (brand, pictorial, and text) in magazine advertisements. That means that objects that did not fall into one of those three predefined categories were not part of any AOI.

An important related question is what exactly constitutes an object. This is, in fact, a much broader philosophical and psychological issue than we can address here. Feldman (2003) argues that a visual object is not simply defined by the physical stimulus, but rather by the observer’s perceptual and cognitive processes. The perceptual component is illustrated by Oliva and Torralba (2006, their Fig. 2) and Fredericksen, Bex, and Verstraten (1997). Nevertheless, it has important implications for the creation of AOIs. For instance, what is the appropriate level of detail when producing AOIs? Consider the panda search task (Fig. 4). AOIs could be defined for entire faces, or for smaller objects such as for eyes, noses, and mouths. As a general guideline, if the research question does not require highly detailed AOIs (such as individual face parts), we recommend keeping them coarse (whole face). Coarse AOIs are more robust for inaccurate eye tracking data (see the next section). However, detailed AOIs for, for instance, the eyes are required if the research question is whether eyes are specifically fixated during the search for the panda.

#### Should the AOIs be small or large?

As noted in the section on crowding, in a sparse stimulus, an observer can fixate relatively far away from an object and still perceive it. If you are not convinced, try applying the naked-eye method to a sparse stimulus. Despite the minimal crowding in sparse stimuli, observers often fixate on the objects in a display (e.g., Nuthmann et al., 2019), rather than the space between objects. In a sparse stimulus, we recommend making the AOI as large as possible (Hessels et al., 2016b) because it has no downside. However, we can think of at least one exception to this rule. In gaze interaction (using a person’s gaze as an input method to interact with a computer interface), the control screen might be a sparse stimulus, consisting of only four buttons interspersed with text. The AOIs should be appropriately sized for the buttons, because we don’t want the buttons to activate without us looking directly at them.

In dense stimuli, crowding is typically more pronounced than in sparse stimuli (Fig. 3A). This means that the observer must fixate closer to objects in order to identify them. Generally, AOIs will be smaller in dense stimuli; however, crowding also depends on the similarity between the target and its background, context, or flankers (Fig. 3B). In such dense scenes, targets may differ in conspicuity. To illustrate with an extreme example: if the panda in Fig. 4A were colored orange or pink, it would stand out more prominently against the black-and-white black-metal artists, thereby resulting in a large conspicuity area. A conspicuous, task-relevant target could therefore be assigned a larger AOI than an inconspicuous one.

In general, no straightforward recommendation can be made about AOI size in dense stimuli. We refer the reader to the “Perception of an object while fixating next to it” and “The naked eye method” sections. A pilot experiment may also be useful in determining the appropriate AOI size. If the hypothesis involves comparing the same objects across different images or displays, then it normally makes sense to try and keep the AOIs the same size.

Can AOIs be too small? The answer is yes. When inaccuracy – that is, the deviation between recorded and actual gaze positions – is large relative to the size of an AOI, there is an increased risk that fixations will be misassigned, particularly when AOIs are small and located close to one another (Holmqvist, Nyström, & Mulvey, 2012). Such misclassifications can result in missed fixations or false positives during analysis. An illustrative example is that the inaccuracy of eye-tracking data in reading research may mean that a fixation is not only not assigned to the AOI the participant looked at, but also incorrectly assigned to a neighboring AOI.

Holmqvist et al. (2011, p. 223) recommends a minimum AOI size of 1$^\circ$ to 1.5$^\circ$ even for high-accuracy, pupil-based eye trackers, which typically have inaccuracies around 0.5$^\circ$ (Hooge et al., 2025a), meaning that an AOI size of 1$^\circ$ diameter would accommodate a 0.5$^\circ$ offset in any direction. Similarly, Orquin and Holmqvist (2018) emphasizes that AOIs should be sufficiently large to remain robust in the face of measurement inaccuracy. They introduce the concept of capture rate, defined as the percentage of fixations that fall within an AOI. Even for high-accuracy systems such as the EyeLink 1000, they recommend using AOIs with a minimum diameter of 3.2$^\circ$ to achieve an 80% capture rate (see their Table 1). However, we caution readers against taking these values at face value, as they are likely too pessimistic. The capture-rate model is based on several assumptions that we find problematic. First, it assumes fixations are uniformly distributed across the entire AOI, whereas in practice, fixations tend to cluster near object centers. Second, the model treats precision as a source of jitter affecting fixation locations. In reality, precision affects individual data samples, and fixation positions – being aggregates of multiple samples – are much less impacted. As a result, the model’s predictions underestimate actual capture rates. We recommend using the inaccuracy of the eye-tracking data as the absolute minimum margin for AOI definition, in line with the guidance of Holmqvist et al. (2011).

### Creation of AOIs

How can you create an AOI? To our knowledge, there are only two primary methods for defining an area within the stimulus display. The first is shape-based, where the AOI is defined as the outline of an object (Fig. 5B) or a region. This approach may involve a precise contour surrounding the object of interest (as in Russell et al., 2008), a contour with an added margin (see Figure 1 of Auyeung et al., 2015), or simpler geometric forms such as rectangles, ellipses, or closed polygons (e.g., Nuthmann et al., 2019; Tatler, Wade, Kwan, Findlay, & Velichkovsky, 2010).

The second method defines the AOI based on a point, typically placed at the center of the object (Fig. 5H) (Hessels et al., 2016b). During the implementation phase, a surface is defined around this point to serve as the AOI. This method is appealing because points can be selected manually, while the AOI’s shape and size can be programmatically generated. A key advantage is the reduction of subjectivity – particularly avoiding hand-drawn shapes – making AOIs easier to reproduce (see the “AOI reproduction” section). The selection of points can also be automated using software. For example, in studies of gaze behavior on faces, Hessels et al. (2018a); Holleman, Hessels, Kemner, and Hooge (2020); Holleman et al. (2021); Vehlen et al. (2022); Viktorsson et al. (2023) used OpenFace (Baltrušaitis et al., 2016) to detect the centers of the eyes, nose, and mouth. In body perception research, Hessels et al. (2020); Callemein, Van Beeck, Brône, and Goedemé (2019), and Müller and Mann (2021) utilized points generated by OpenPose (Cao, Hidalgo, Simon, Wei, & Sheikh, 2019). Similarly, Gehrer et al. (2024) employed a joint and skeleton model from MediaPipe by Google. All examples discussed here apply both to still images (static AOIs) and video sequences (dynamic AOIs). We will return to dynamic AOIs later in the article.

While AOIs are frequently used, few researchers report on how they were implemented because most eye-tracking researchers rely on dedicated AOI editors (see the next section, *Implementing with an AOI Editor*) or fully automatic AOI production (e.g., Fuhl et al., 2018). However, some researchers create AOIs manually (either by drawing them in a graphics program or defining them as polygons) and then use custom-built software for AOI analysis. The final two sections on the implementation of AOIs provide a relatively detailed explanation of how to conduct AOI analyses using such custom software. While we could have described multiple approaches, we chose to focus on what we consider the most intuitive one: implementing AOIs as binary (black-and-white) images. Two of the authors implemented this method in the 2000s for a marketing research company.

#### Implementation with an AOI editor

After deciding on the nature of the AOIs, AOI implementation may start. Researchers who have access to corporate eye tracker software can use the included AOI editor. An AOI editor is usually a computer program with a graphical user interface that allows AOIs to be drawn. A plus is that these software packages also take care of computing AOI statistics. Examples of commercial software suites with an AOI editor include: SR Research Data Viewer, SMI BeGaze,^6^ Gazepoint Analysis, iMotions, and Tobii Pro Lab (non-exhaustive list). Some of these AOI editors can also import and export AOIs (e.g., as XML files), allowing them to be reused in a new study, in the same study, but for another or the same stimulus, or by another researcher. There is also free eye tracker-agnostic software available, such as Eyetrace (Kübler et al., 2015; Otto, Castner, Geisler, & Kasneci, 2018) or OGAMA (Voßkühler, Nordmeier, Kuchinke, & Jacobs, 2008).

Creating AOIs in AOI editors typically involves drawing geometric shapes – such as squares, circles, ellipses, free-form outlines, or polygons—around objects in visual stimuli (see Fig. 5C), and assigning them meaningful labels (e.g., fork, egg, knife, ketchup_bottle). The implementation of these AOIs (Fig. 5D), as well as the labeling of fixations or gaze samples with corresponding AOI identifiers (Fig. 5E), is handled automatically by the AOI analysis software. In short, with the appropriate tools, defining AOIs and analyzing eye tracking data has become a straightforward task. As an example, here is a link to YouTube video tutorials using SR Research Data Viewer for the creation of static AOIs (https://www.youtube.com/watch?=YOTpfiN5RlM).

However, not all researchers have access to corporate software such as Tobii Pro Lab, which requires a license. For those working with Tobii eye trackers, we recommend considering Titta (Niehorster, Andersson, & Nyström, 2020), a free and open-source toolbox^7^ for controlling Tobii eye trackers from Python and MATLAB. Titta includes several demo analysis methods, one of which supports AOI analysis. In fact, most of the routines are generic and not specific to data from Tobii eye trackers. What needs to be done is to take the output of any eye tracker, transform it into a format that Titta takes as input. However, it does not provide an AOI editor. AOIs must be created externally using image editors with pixel-based output, such as Photoshop, GIMP (the freely available open-source option) and PowerPoint. The section below, “Implementing black and white binary AOIs with a drawing program” section, outlines a step-by-step method for creating AOIs compatible with Titta. We include this guide as it may also benefit researchers developing their own AOI analysis routines (see the “Custom software for assigning AOI labels to gaze data” section below).

#### Implementing black and white binary AOIs with a drawing program

The goal is to produce a black-and-white (1-bit) PNG file for each AOI. For each stimulus display from the experiment (e.g., ketchup, see Fig. 5A), create an AOI folder named after the original stimulus display (e.g., ketchup). In this AOI folder (see Fig. 5C and D), place a copy of the display image file (e.g., copy_of_ketchup.png). You should use a copy to ensure that the AOI mask images have the same resolution as the original stimulus image. Open this image file with a pixel-based image editor (e.g., Photoshop, GIMP). You can now create the first AOI (e.g., egg) by removing or erasing (i.e., making white) the part of the display that corresponds to the AOI (egg, see Fig. 5D). Everything that is not part of the AOI should be made black. Name it *egg.png* and save this image file as a black-and-white (1-bit) image file^8^ in the AOI folder. One AOI may consist of multiple white areas (a distributed AOI,^9^ see Holmqvist et al., 2011, p. 210). If you need four AOIs as in Fig. 5B), repeat this whole process four times. We suggest having four copies of the stimulus display with the names of the AOIs prepared in advance (fork, egg, knife, and ketchup bottle). There are several ways to do this. If your image processing software allows it, you can also perform the above steps using four layers and export them later as separate PNG files. To simplify the statistical analysis, we recommend using non-overlapping AOIs so that each gaze sample or fixation belongs to at most one AOI. This approach does not allow an AOI (for example, a face) to contain smaller AOIs (such as the eyes, nose, and mouth). Researchers who wish to perform AOI analyses at different levels of detail can accomplish this by running multiple AOI analyses sequentially.

**Fig. 6:**
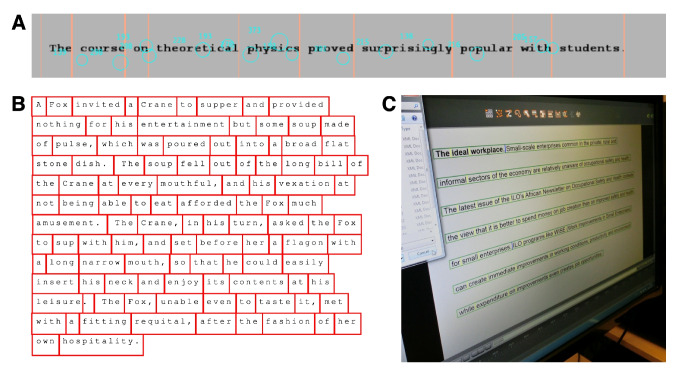
Full segmentation of text into word AOIs. (**A**) A single-line sentence with AOIs created using SR Research Experiment Builder and visualized with SR Research Data Viewer. *Orange vertical lines* indicate word boundaries, with fixations from one reader overlaid as *circles*. (**B**) Multi-line text segmentation of a story presented in Nuthmann and Henderson (2012). Word boundaries spanning multiple lines were created using SR Research software and visualized in MATLAB, showing AOIs over a full-page layout. (**C**) Word AOIs produced with BeGaze. Note the narrow vertical size of the AOIs

#### Custom software for assigning AOI labels to gaze data

The recipe below provides a framework for implementing a custom AOI analysis on gaze data from any eye tracker. This approach is especially valuable when identical AOIs and analysis procedures need to be applied across datasets collected from different eye trackers – a common scenario in labs equipped with systems from multiple manufacturers. Not all researchers, however, have the time, interest, or expertise to develop their own AOI analysis scripts. In such cases, this recipe can be handed off to a departmental technician or programmer for implementation. This offers a practical solution for labs seeking a consistent AOI analysis method across different systems. The implementation of the AOI analysis described below (see also Fig. 5E) is tailored for analyzing static stimuli (e.g., photos, advertisements, or screenshots), using the black-and-white AOIs introduced in the previous section (Fig. 5D). Note that Titta offers an implementation of this strategy that can also be handed to the programmer (there is no need to implement this from scratch, only a different read-in function to support data in a different format should be required).

AOI labeling of fixations (or single gaze samples) is a simple job for a computer. The script should check the color of the pixel at each fixation (or gaze sample) coordinate from the AOI file (e.g., egg.png). If the pixel is white, the fixation (or gaze sample) is assigned to the AOI *egg* and labelled *egg*; if black, it remains unlabeled (or keeps the default label *none*). This should be repeated for all present AOIs (fork.png, egg.png, knife.png, and ketchup_bottle.png) in the AOI folder (ketchup).

Be sure to account for gaze points that do not fall within any AOI by assigning them to a *none* or *whitespace* AOI (Holmqvist et al., 2011, p. 206). We recommend assigning the label *none* to all fixations or gaze samples by default. Fixations that occur outside the screen area can be labeled as *offscreen*. Once fixations or gaze samples are labeled, the start and end times of each fixation can then be used to compute AOI-related statistics, such as fixation count, time to first fixation (TFF), visit count, and the percentage of time spent within each AOI.

## Automatic AOI production methods

In this section, we focus on the use of automatic full segmentation and annotation methods to assign objects and regions within a stimulus to AOIs. This method will be illustrated with examples from reading research and from the field of scene perception.

### Full segmentation for AOI production in reading

In reading research, AOIs are commonly employed due to the structured nature of stimuli and tasks that facilitate their use (e.g., Godfroid, 2019, Chapter 6.1).

Fixations during reading can be analyzed at different levels, including paragraphs (e.g., Knoop-van Campen, Ter Doest, Verhoeven, & Segers, 2022), sentences (e.g., Liversedge et al., 2024), words (e.g., Kliegl, Nuthmann, & Engbert, 2006), and even individual letters (e.g., Nuthmann, Engbert, & Kliegl, 2005). At the coarsest level (paragraphs), AOIs can be drawn manually as rectangles (see Fig. 1 in Knoop-van Campen et al., 2022). In reading experiments where selected target words are embedded in sentences or text passages (e.g., Rayner & Duffy, 1986), AOIs can be created either manually or automatically.

In quasi-experimental studies, eye movements are recorded while participants read a corpus of natural text, consisting of individual sentences or passages (e.g., Jakobi, Kern, Reich, Haller, & Jäger, 2025). A widely used AOI-production method in such studies is full segmentation at the word and/or letter level. For visualizations of this approach, see Fig. 6 in the present article, Busjahn and Tamm (2021, Fig. 1), who investigate code reading, and Hooge et al. (2025b, Fig. 3).

For users of EyeLink systems with access to SR Research software (Experiment Builder and Data Viewer), AOIs can be automatically created around regions of text.^10^ These AOIs can then be used in Data Viewer to extract common eye-movement measures typically employed in reading research. As a first example, we use data from a reading study involving single-line sentences (see also Fig. 3 in Hooge et al., 2025b). Figure 6A displays a trial-based visualization from Data Viewer, where orange vertical lines indicate word boundaries. The space character is included as part of the AOI for the following word (e.g., McConkie, Kerr, Reddix, & Zola, 1988). The second example involves a story adapted from Aesop’s fable *The Fox and the Crane*, as used by Nuthmann and Henderson (2012). Figure 6B visualizes the word boundaries that were created using SR Research software. Overall, while the automatic segmentation is not perfect, it proves sufficient for most practical purposes.

Automated segmentation can also be achieved using open-source tools or optical character recognition (OCR) engines. Figure 6A and B display text that was set in a fixed-width or non-proportional font (i.e., all letters have the same width) to control for visual spacing. This uniform spacing facilitates the automated segmentation of text into words and characters using scripting languages like R (see Hooge et al., 2025b, for details).

By contrast, the text shown in Fig. 6C was set in a proportional font, in which character widths vary. This variability makes automated segmentation more challenging and has motivated the use of alternative approaches. One such approach is OCR, which enables the automated detection of text regions and their content within digital images. In eye-tracking research, OCR allows for automatic creation of AOIs by identifying both the position and content of words or characters in text stimuli (e.g., Le, Nguyen, Le, Nguyen, & Ngo, 2023). While tools like Eyeflow Studio (Culemann & Heine, 2024) are beginning to incorporate automated AOI-detection capabilities using OCR engines such as Tesseract (Smith, 2007), OCR-based approaches for AOI creation in reading research remain largely exploratory rather than standard practice.

How can reading researchers adapt AOIs to the eye-tracker inaccuracy? To begin with, they can minimize the impact of inaccuracy by designing studies that reduce it. For example, measurements taken near the center of the screen tend to have higher spatial accuracy and lower measurement error. Analyzing eye movements at the finest level, the letter, requires an eye tracker with high accuracy and precision (e.g., a Dual Purkinje Image eye tracker in McConkie et al., 1988) and/or sufficient data for reliable results.

There is limited flexibility in adjusting the AOI size. Increasing font size and spacing between words and characters can reduce the impact of eye-tracker inaccuracy, but only within limits. Larger fonts reduce how much text fits on the screen, and wider spacing may disrupt reading (Paterson & Jordan, 2010). With text AOIs, it is not possible to add horizontal margins. However, since readers do not always fixate precisely on the line of text (Nuthmann, 2013), adding a vertical margin is recommended. When using multi-line text, line spacing should be larger than in typical printed text (see Hooge et al., 2025b). Neither Fig. 6B nor C fully follows these guidelines. In Fig. 6B, line spacing (1.5) is relatively tight, which may complicate fixation-to-line assignment; note, however, that the authors did not intend to perform word-based analyses. In Fig. 6C, although the inter-line distance is larger, no vertical margin has been added.

**Fig. 7:**
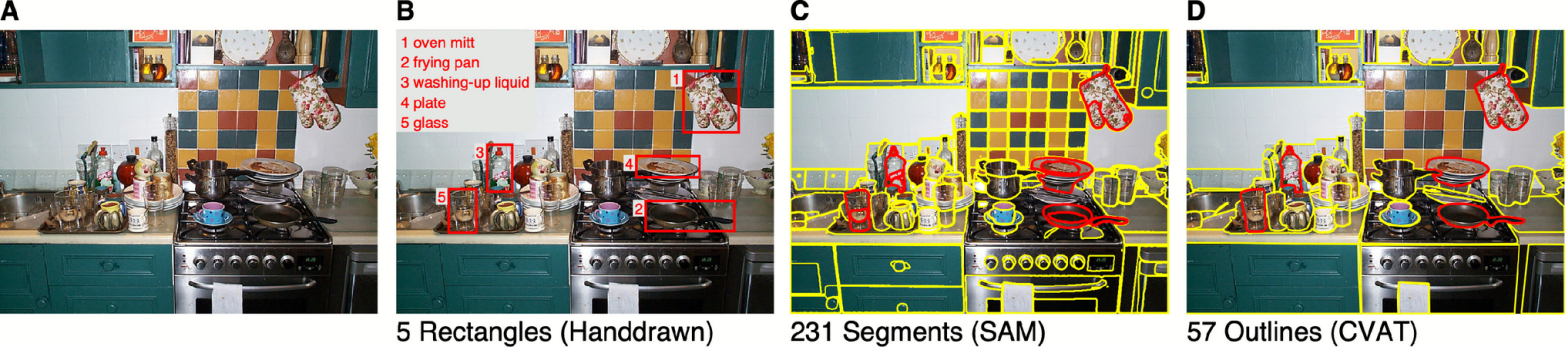
AOIs in scene perception research. (**A**) Example image of a real-world scene. (**B**) Partial manual object annotation with *rectangles* (adapted from Nuthmann et al., 2020). (**C**) Complete automatic segmentation with the Segment Anything Model, which served as a basis for the manual annotations in panel D. (**D**) More complete manual object annotation using polygons created with the CVAT software. In panels C and D, the five objects annotated in panel B are shown in *red*; all other objects are in *yellow*. The depicted photograph was taken by George L. Malcolm (Nuthmann & Faul, 2026)

### Image segmentation and object annotation

According to Henderson and Hollingworth (1999, p. 144): “a scene is a semantically coherent (and often namable) view of a real-world environment comprising background elements and multiple discrete objects arranged in a spatially licensed manner”. The scope of AOIs within naturalistic real-world scenes depends on the research question: AOIs may correspond to a single predefined critical object (e.g., Cornelissen and Võ, 2017), a set of objects meeting specific selection criteria (e.g., Nuthmann et al., 2020), or an exhaustive annotation of all scene elements (e.g., Hayes and Henderson, 2021; Malcolm et al., 2014).

A practical approach to achieving more complete object annotation with polygons is to combine powerful segmentation models with advanced annotation tools. For example, the partial object annotations using rectangles in the study by Nuthmann et al. (2020), as illustrated in Fig. 7B, have since been extended using the Segment Anything Model (SAM, Kirillov et al., 2023) and the Computer Vision Annotation Tool (CVAT, https://www.cvat.ai). In automatic mode (i.e., without prompts), SAM produces complete, detailed, and often overlapping segmentations to offer a broad set of candidate regions (Fig. 7C). These segmentations were then carefully curated and refined by a human annotator using CVAT (Fig. 7D), a state-of-the-art annotation platform that offers free access to core image and video labeling features for individual users.

When using more complete stimulus annotations, specific challenges emerge. Particularly small objects may be excluded because limitations in the eye tracker’s spatial accuracy and natural variability in eye-movement control can make it difficult to reliably assign fixations to these objects (see also “Should the AOIs be small or large?” section).

Various practical issues can arise when producing AOIs (e.g., with image processing tools that were not directly developed to design AOIs). For example, when two polygons share a boundary, fixations or gaze samples that fall on that boundary cannot be reliably assigned to either polygon. This is effectively the same problem as overlapping AOIs. Therefore, shared boundary segments between polygons should be avoided, just as overlapping AOIs should be. In case that boundary sharing cannot be avoided, a decision rule for boundary pixel assignment (i.e., rolling a dice) to the AOI may offer a solution.

Another example of a practical problem is when there are small gaps between AOIs. In that case, fixations may remain unassigned to any AOI. A solution is the use of the *none* AOI, which is also mentioned in the "Custom software for assigning AOI labels to gaze data". Another approach is to assign a fixation to the nearest AOI.

The possibilities in the field of automatic AOI generation and annotation may seem limitless. However, we encourage anyone working with these tools to critically evaluate the automatically generated AOIs (e.g., considering aspects such as borders, minimal AOI size, and spacing between AOIs). Figure 7C and D show how the segments produced by SAM were refined by a human annotator using CVAT to meet the specific requirements of the study. Whether such tools save time will depend on the context and must be judged individually.

## Dynamic AOIs

Dynamic AOIs are necessary for analyzing eye-tracking data involving moving stimuli, such as videos, animations, or scene camera recordings from a wearable eye tracker. Dynamic AOIs differ from static AOIs in that they (1) may change in position, size, or shape over time to match moving elements within the stimulus, and (2) may appear and disappear at specific moments. Nowadays, eye tracking in dynamic environments is far more common than it used to be. This is mainly due to significant technological advancements in wearable eye trackers, which have become much more affordable and user-friendly.

We want to distinguish between two types of dynamic environments in eye tracking, each presenting unique challenges for AOI production. The first type involves eye tracking of moving stimuli on a screen (e.g., video clips, TV commercials, movies). This type of dynamic visual stimulus is identical for all participants, so one set of AOIs for the dynamic stimulus is sufficient. That said, this is far from easy, manually creating AOIs for films might be a massive task, even when using keyframes and interpolating between them. In practice, manual dynamic AOI creation is only feasible for short clips like TV commercials.

The second type involves video from the scene camera of a wearable eye tracker. The major difference from the first category is that these videos are unique for each participant. In our view, manually producing AOIs for such data is an unmanageable task. Even when the environment is not changing, and there is a single stimulus of interest (such as a face when two people are seated and conversing), manually drawing AOIs would be very time-consuming and difficult to reproduce due to variations in head position and distance. Researchers typically address this problem by not producing AOIs at all. Instead, they manually annotate the video overlaid with gaze data (e.g., Foulsham, Walker, & Kingstone, 2011; Hessels et al., 2022; Rogers, Speelman, Guidetti, & Longmuir, 2018). The AOIs are then made implicit by the annotator, who decides, potentially informed by knowledge of the eye tracker’s inaccuracy, how far a gaze point can deviate from an object and still be counted as a fixation on that object. While manual coding remains common in mobile eye tracking, it also involves judgments that could be subjective. For example, when asked to code fictional eye tracking data, participants are more likely to allocate a fixation to a person than to an object, even when it is the same distance away (Dawson, Kingstone, & Foulsham, 2021).

In the next section, we present a practical solution for the AOI analysis of wearable eye tracking data that may be applied to for example, small or well-known or very structured environments (as in Dik, Hooge, van Oijen, & Siersema, 2016; Ouzts, Duchowski, Gomes, & Hurley, 2012; Pfeiffer & Renner, 2014).

**Fig. 8:**
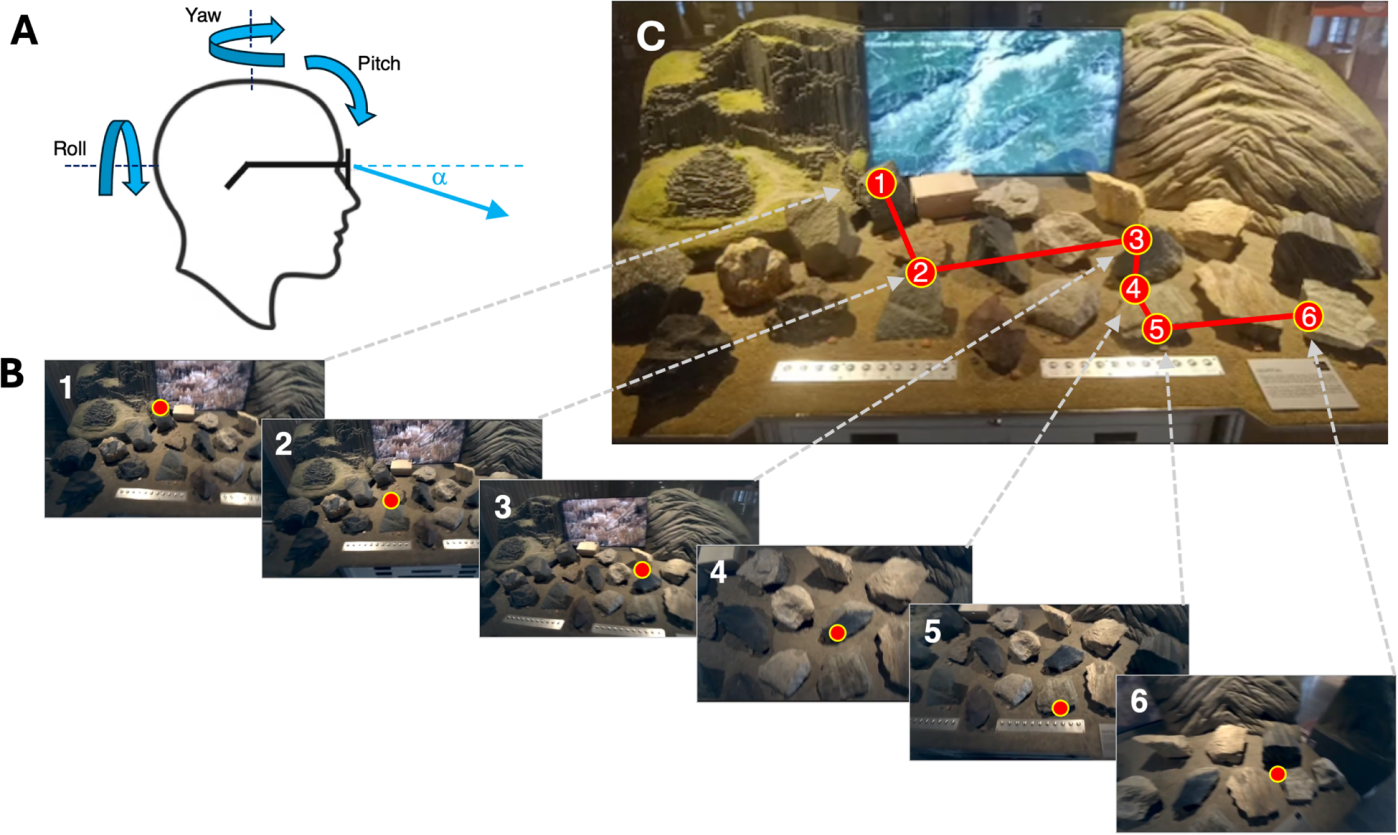
Gaze in head and world reference frames. (**A**) The wearable eye tracker is attached to the head. It contains at least two cameras: one camera to estimate eye orientation relative to the head ($\alpha$), and one to record the world – the scene camera. When people look around, they usually rotate their eyes and head. (**B**) As a consequence of these head movements, the scene camera image may vary over time. The six panels show individual fixations (*red circles with yellow borders*) overlaid on the changing scene camera image. These six images show fixations in the reference frame of the head. (**C**) The observer’s six fixations mapped onto a photo of the display case. These fixations are shown in the reference frame of the display case (the world). When transformed to a world-based reference frame, one can perform a standard AOI analysis. The pictures were provided by Stanislav Popelka

**Fig. 9:**
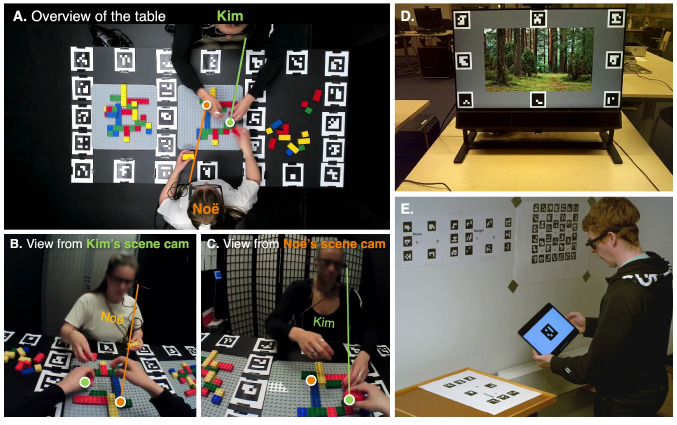
Fiducial markers. All panels in this figure contain the black-and-white ArUco markers. The unique ArUco markers are essential for transforming the eye tracking signal from the head-centric frame of the wearable eye tracker to the reference frame of the table (A, B, and C), screen (D), wall, corkboard, and iPad (E). (**A**) This is the view from an overhead ceiling camera capturing a table with two participants (Kim and Noë), each wearing eye-tracking glasses. Together, they perform a LEGO model building task (as in Hessels et al., 2023; 2025a). The green gaze point represents where Kim is looking, while the orange gaze point shows Noë’s gaze. You may wonder why the lines to the gaze points come out of the side of the eye tracker. This is because in this example we used the Pupil Labs Pupil Invisible eye tracking glasses, which has the scene camera on the left side. The gaze points on the table (A) are mapped versions of the gaze data from Kim’s (B) and Noë’s (C) scene cameras. This mapping was made possible by the numerous unique ArUco markers placed on the table. In the overview of the table (A), it appears as though Noë is looking at Kim’s hand. However, in the views from both Kim’s and Noë’s scene cameras (B and C), it appears that Noë is looking underneath Kim’s hand. This discrepancy arises because the gaze points are projected onto the plane of the table. (**D**) This is a monitor equipped with ArUco markers. This setup could be used to enable screen-based eye tracking using wearable eye-tracking glasses. (**E**) The lab of Diederick Niehorster. He is looking at an iPad with an ArUco marker on it. The pictures originate from https://github.com/dcnieho/gazeMapper. A, B, and C were taken during the data collection of Hessels et al. (2023)

### Circumventing the trouble: Transformation to a world-based frame of reference

Commercial wearable eye tracking became more accessible and popular after 2010 with 1) the introduction of the Tobii Glasses and the SMI ETG that were both much smaller than, for example, the previous generation SMI helmet-mounted eye trackers (Gullberg & Holmqvist, 2006, Fig. 3) and 2) the introduction of the much cheaper Pupil Labs wearable eye trackers in 2014. These wearable eye trackers include cameras that record the eyes (eye camera) and a forward-facing camera that captures the world (scene camera). The output signal is the pixel being looked at in the scene camera’s video. As such, the wearable eye tracking glasses provide the orientation of the eye relative to the head. Most researchers who use wearable eye trackers are usually not interested in eye orientation relative to the head, but rather in the gaze point in the world. For example, they may study fixations on store products (e.g., Gidlöf, Wallin, Dewhurst, & Holmqvist, 2013), where people look while assembling a tent (Sullivan, Ludwig, Damen, Mayol-Cuevas, & Gilchrist, 2021) or how gaze behavior during locomotion compares between virtual reality and the real world (Drewes, Feder, & Einhäuser, 2021).

It would be great to be able to perform a standard AOI analysis on eye-tracking data from eye-tracking glasses. These AOIs often concern objects located in the real world. Since the eye tracking glasses are attached to the rotating and translating head, the relationship between gaze direction and objects is not directly apparent.^11^ Determining which objects in the world the observer is looking at is therefore typically done by manually annotating the scene camera footage with an overlaid gaze point (e.g., Benjamins, Hessels, & Hooge, 2018; Foulsham et al., 2011; Gidlöf et al., 2013; Laidlaw, Risko, & Kingstone, 2012; Maran, Hoffmann, & Sachse, 2022). Another method is the use of an abstract reference image for manual annotation of fixations (Trefzger, Blascheck, Raschke, Hausmann, & Schlegel, 2018, Fig. 2). Hoffmann et al. (2024) had human annotators determine where the participant was looking at another person, using hand-drawn AOIs of the face, eyes, body, and background on a frame from the scene camera of the wearable eye tracker. Manual annotation is tedious, costly, subjective, and labor-intensive (e.g., 9 min of annotating for one minute of eye tracking data, Rogers et al., 2018).

An alternative method is to let computers automatically detect objects in the scene camera footage and use those to generate dynamic AOIs. We will return to this approach later in the “Implementing dynamic AOIs” section. However, we might also consider circumventing the problem entirely. In general, an effective way to solve a difficult problem is to apply a transformation that moves the problem into a representation or space where it becomes easier to solve or compute. What if we could transform eye tracking data from a head-centered reference frame to a world-based reference frame?

#### Reference or assisted mapping

We describe two methods for mapping fixations from the head-centered reference frame to the world-centered frame of reference found in commercial software. The first method is based on a form of image recognition. *Tobii Assisted Mapping* (as opposed to manual mapping) allows researchers to transform head-centered gaze data from Tobii glasses by using snapshots containing key objects and matching them to specific areas in the environment. Also, this automatic mapping can be complemented by the manual mapping approach, so that if one disagrees with the automatic mapping, one just re-maps that specific point. Also, if the software provides a score of how well the mapping matched, the researcher can prioritize manually remapping the lowest scores. *Tobii Assisted Mapping* is achieved through video recordings from the scene camera (Fig. 8B). The resulting static world-based representation (Fig. 8C) can then be used for standard static AOI analysis and heatmap production (and other visualizations). Pupil Cloud software offers a comparable functionality for Pupil eye-tracking glasses (*Reference image mapper enrichment*), enabling the generation of heatmaps (e.g., aggregated across multiple participants) or gaze overlay videos on top of a static reference image. This representation is created using the scene camera video, a reference image, and a reference video. Researchers can create the reference video by scanning the object or area of interest using the eye tracker’s scene camera.

#### Fiducial marker technology

The second method relies on reference markers, known as fiducial marker technology (e.g., ArUco markers, Garrido-Jurado, Muñoz-Salinas, Madrid-Cuevas, & Marín-Jiménez, 2014). In this approach, the experimenter places markers of known size at predetermined locations in the environment (Fig. 9). Some software packages bundled with commercial wearable eye trackers (e.g., Pupil Capture and Pupil Player with proper plug-ins), the OpenCV library and the MATLAB Computer Vision Toolbox can detect fiducial markers in the scene camera image. This enables the transformation of eye-tracking signals from a head-referenced coordinate system to a world-based reference frame. Examples include: Tabuchi and Hirotomi (2022), who used fiducial markers to analyze wearable eye-tracker data collected during cooking tasks; De La Hogue, Mouratille, Causse, and Imbert (2024), who developed ArGaze (a software library for gaze analysis using fiducial markers) and applied it to eye-tracking data recorded in a cockpit; Bykowski and Kupiński (2018), who attached an ArUco marker to a phone, enabling the transformation of head-centered gaze coordinates from eye-tracking glasses to the reference frame of the phone’s screen.

Niehorster, Hessels, Nyström, Benjamins, and Hooge (2025a) utilize the ArUco marker technique in gazeMapper, a tool designed for the automated transformation of head-centered gaze data to planes (e.g., a table, a screen, a wall, a dashboard, see Holmqvist et al., 2011, p. 208). A major advantage of gazeMapper is that it is eye-tracker agnostic, allowing data collected from different devices^12^ to be analyzed in a standardized way (by a static AOI analysis). GazeMapper also facilitates experiments involving multiple participants using various eye-tracking systems within the same setup (e.g., three participants playing a card game, one wearing Tobii Pro Glasses 2, another wearing the SeeTrue ST ONE, and one wearing the Pupil Neon).

The main downside of fiducial marker technology is that the physical environment must be equipped with visible markers. For some studies, this poses no issue (e.g., Hessels et al., 2023, 2025a), but for others, it does. For instance, imagine how a museum curator might react if you suggested attaching eight to twelve ArUco markers next to each painting for an eye-tracking study. A potential solution to this visible-marker problem is the use of infrared ArUco markers, as introduced by Ayala et al. (2024), which are only visible to cameras sensitive to infrared light, but appear black to human observers. Another example is social interactions, where it is likely undesirable to add markers around or within faces when evaluating eye contact or attention to faces (e.g., Hoffmann et al., 2024). However, the application of fiducial marker technology has its limitations. Some environments are too large and dynamic for the use of fiducial markers (e.g., a football field or a traffic roundabout full of moving cars). In general, fiducial marker technology is best suited for laboratory settings or simulators.

Once eye-tracking data is mapped onto a plane (e.g., a table, screen, phone, a wall full of pictures, or the cockpit of a plane), a static AOI analysis, as described in “Static AOIs” section, can be applied.

### Dynamic scenes

The simplest approach to annotating a stimulus movie with AOIs is to treat the movie as a collection of independent frames. These frames can then be handled in the same manner as described in “Static AOIs” section. To clarify, let us illustrate a simple AOI production case with an example. Suppose we are interested in visual information processing during lip reading. At which part of the face does an observer look while lip-reading? To test this, participants watch short clips of an actress speaking words while being eye tracked. To create AOIs on the face, we could apply the method described by Hessels et al. (2018a). For each frame of the movie featuring the actress, we use OpenFace (Baltrušaitis et al., 2016) to determine the centers of the eyes, nose, and mouth. These centers form the basis for the AOIs (as shown in Fig. 5I). The clip for the lipreading experiment is highly suitable for automated AOI processing without human interference due to the following factors:

- Static Camera: The camera remains fixed in position, with no translational or rotational movement. This means that objects that do not move in the real world remain in place in the film and also do not change in size in the camera image.
- Minimal Subject Motion: The actress exhibits very limited movement throughout the clip.
- Consistent Visibility: She remains within the frame from the beginning to the end of the video.
- Isolated Subject: No other individuals appear in the scene, ensuring a clear and unambiguous focus.

If the movie clip contains minimal motion (e.g., an actress who remains mostly still, with only subtle facial movements while speaking), an approach utilizing static AOIs instead of dynamic AOIs may be viable. Whether this approach is satisfactory, however, is an empirical question. Researchers could explore this possibility in a pilot study during the preliminary phase of their investigation (Hessels et al., 2025c).

A more complex situation concerns the footage of the scene camera of a wearable eye tracker. A wearable eye tracker’s scene camera may capture video in controlled and contained environments like a supermarket, operating room, or aircraft cockpit. As an example, Friedrich, Rußwinkel, and Möhlenbrink (2017) present a guideline for integrating dynamic areas of interest in the context of aviation research (e.g., coordinate mapping, object tracking, AOI definition, applications), illustrated with a study in an air traffic controller environment. However, producing dynamic AOIs becomes substantially more difficult when the environment is expansive and features unpredictable events or unfamiliar objects – for instance, scene camera recordings of a cyclist navigating a city or a soldier in a field training exercise.

The complexity of dynamic AOI production increases when analyzing longer videos in which objects appear and disappear, and both relevant and irrelevant elements coexist within the scene. Real-world footage – such as news segments, TV commercials, or short films – often consists of multiple scenes, further adding to the challenge. In the next section, we will describe several methods available for implementing dynamic AOIs.

### Implementing dynamic AOIs

One of the earliest techniques for generating dynamic AOIs is the keyframe method. In this approach, AOIs are manually drawn on selected keyframes within a video or animation. Interpolation is then used to estimate the AOI’s position and shape across the intermediate frames. Finally, gaze data is assigned frame-by-frame onto the resulting AOI regions. This method is supported by commercial software such as Tobii Pro Lab, iMotions, and SR Research Data Viewer (see Körner, Faul, & Nuthmann, 2023). Free alternatives also exist, including OGAMA (Voßkühler et al., 2008) and DynAOI (Papenmeier & Huff, 2010). Despite software support, the process remains relatively labor-intensive.

An alternative is to combine the keyframe technique with automatic object recognition (e.g., STEGO, Körner, Faul, & Nuthmann, 2024) or with the point method (see Fig. 5I) to produce circular dynamic AOIs (Fichtel et al., 2019).

Is the keyframe technique still necessary? With sufficient computational resources, automated object recognition can be applied to every frame of a video. For example, Hermens (2024) uses the YOLO (You only look once) algorithm to detect an object of interest (a surgical tool) in a video. This computer vision algorithm is pre-trained on object recognition and can then be trained to detect a particular target (given a relatively small number of labelled examples). Several studies have already adopted the object recognition approach (Bonikowski, Gruszczyński, & Matulewski, 2021; Eraslan et al., 2020; Kang, Mandal, Crutchfield, Millan, & McClung, 2016; Lagmay et al., 2022; Mercier, Ertz, & Bocher, 2024; Nguyen, Vrzakova, & Bednarik, 2016; Wolf et al., 2018; Zhang, Yuan, Chen, & Liu, 2018). All except Eraslan et al. (2020) and Zhang et al. (2018) implement object tracking across frames to maintain continuity in AOI assignment.

The technology is advancing rapidly. For instance, SAM 2 enables segmentation of indicated objects throughout a video without having to first be trained by the researcher, and even enables full segmentation (Ravi et al., 2024, see https://github.com/facebookresearch/sam2). A key advantage of full segmentation is that it facilitates the analysis of longer and more complex dynamic scenes while reducing human bias (although the model may be biased too). However, limitations of full segmentation remain (see the “Automatic AOI production methods” section): object detection may be inaccurate, and algorithms often make implicit decisions, for example, regarding how to handle occlusion or object permanence.

## Discussion

In the discussion, we will explore two main topics. The first is *Advances and outlook for AOIs in eye-tracking research*, where we will: critically evaluate the implications of automated generation and labeling of AOIs, explore the concept of three-dimensional AOIs, and question whether AOIs are still necessary in contemporary research. The second topic focuses on *Common Issues with AOIs*, such as AOIs derived from gaze data, the choice between basing AOI analyses on fixations versus gaze samples and Ethical issues with AOI production.

### Advances and outlook for AOIs in eye-tracking research

#### About segmentation of whole scenes and movies

Image processing tools have become so powerful that researchers can now automatically segment complex scenes and video sequences into numerous AOIs (e.g., Fuhl et al. 2018; Ravi et al., 2024; Richtsfeld, Mörwald, Prankl, Zillich, & Vincze, 2012), which can also be automatically labeled (e.g., Alinaghi et al., 2024; Deane et al., 2023; Li, Socher, & Fei-Fei, 2009). The benefits of full automatic segmentation are evident: it significantly reduces manual effort for researchers, and makes feasible certain projects that were previously out of reach. Examples of successful approaches include the use of OpenFace (Baltrušaitis et al., 2016), which generates facial markers that support the definition of face AOIs (Duchowski et al., 2019; Hessels et al., 2018a; Vehlen et al., 2022), and OpenPose (Osokin, 2018), which provides skeletal joint positions that enable the construction of body AOIs (Gehrer et al., 2024; Hessels et al., 2020; Müller & Mann, 2021).

**Fig. 10:**
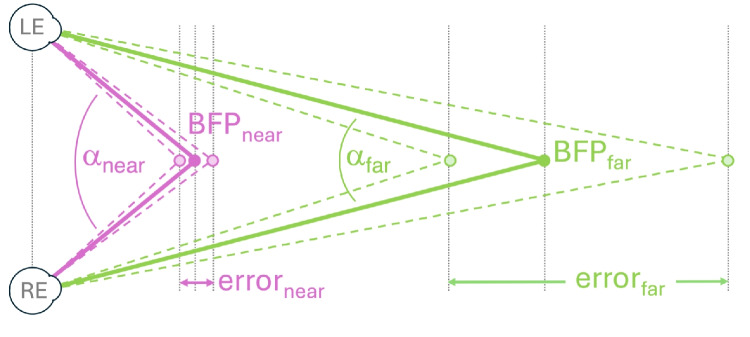
The binocular fixation point (BFP) and inaccuracy. *LE* - left eye; *RE* - right eye; Alpha - vergence angle. A fixed inaccuracy (*dashed lines*) in eye orientation leads to a much larger error in the distance to the BFP during far fixation (*green*) than during near fixation (*pink*)

A critical question arises when we consider the ‘I’ in ‘AOI’: whose interest does it represent, the researcher’s or the algorithm’s? Will the creation of static and dynamic AOIs soon rely entirely on automated image segmentation tools? We doubt that, as automated full segmentation and annotation also have their drawbacks. Below is a non-exhaustive list.

- Fully segmenting visual scenes is not a general solution to the problem of designing and creating AOIs. In fact, it merely postpones the decision-making process about the appropriate AOIs, or worse, it avoids this crucial decision altogether.
- What is an object? Automatic object recognition, segmentation, or labeling may implicitly impose a particular spatial scale for object size (see the earlier example involving faces, eyes, noses, and mouths). In the context of the observer’s task, not every object is necessarily relevant.
- It is also not known how an algorithm will handle occlusion. What does an algorithm do with a cow behind a fence? Is it three pieces of fence and four pieces of cow? Or is it one cow? But then what happens to the fence?
- No more AOIs should be created than are necessary to answer the research question. For some experiments, one AOI per stimulus display suffices.
- It does not take eye tracking data quality into account. If the entire scene is divided into AOIs, at least in dense displays, the resulting AOIs may become too small relative to the inaccuracy of the eye-tracking data (Holmqvist et al., 2012; Orquin, Ashby, & Clarke, 2016; Orquin & Holmqvist, 2018).
- Some AOIs that are important for the research question (and task for the participant) should/could be made larger based on their conspicuity (see the section above on “Perception of an object while fixating next to it” section). In dense displays, this might be not feasible because full segmentation for AOI assignment leaves no (or not enough) available space.
- The question is whether all objects in the scene are relevant to the participant’s task, or whether they are simply part of the background. Consider, for example, a wealthy observer searching for their yellow Ferrari in a parking lot full of Ferraris. Top-down selection in visual search (Cave & Wolfe, 1990; Malcolm & Henderson, 2010; Theeuwes, 2010; Zelinsky, Zhang, Yu, Chen, & Samaras, 2005) effectively ensures that the red Ferraris are not even potential saccade targets for this observer (in other words, they are part of the task-irrelevant background).
- The size of the AOI may depend on the specific task (e.g., detection or identification) given to the participant. This can be illustrated with a traffic-related task. Drivers who need to *detect* a speed limit sign can do so when fixating at much greater distance from the sign than is required to *identify* whether the sign indicates a speed limit of 30 km/h or 40 km/h.

However, automatic AOI assignment may serve as a first step in AOI design. In a second phase, particular AOIs could be enlarged, reduced, removed, or merged (see the “Automatic AOI production methods” section). The list above could be used to develop an application that takes all the mentioned points into account when automatically generating AOIs.

#### What about volumes of interest (VOI)?

Gaze behavior in three dimensions presents a compelling application in VR, AR, and real-world eye tracking. For instance, consider how individuals visually explore a sculpture (as in Stein, Jossberger, & Gruber, 2022). Similarly, how do people allocate their gaze when interacting with a virtual 3D environment? Or where do dentists direct their attention when filling a cavity?

By intersecting the gaze direction vector with an object in the world, a gaze point on that object can be determined. Some eye trackers record the gaze direction of both eyes independently. With data from such a binocular eye tracker, it is theoretically possible to determine the binocular fixation point (BFP), the point in 3D where the two gaze direction vectors intersect.

The location of the BFP in three dimensions can be especially relevant for studying attention in real-world settings, as well as for eye tracking in AR and VR environments. Rather than relying on two-dimensional AOIs, the BFP opens up the possibility of using Volumes of Interest (VOIs), which represent three-dimensional regions of attentional focus. The BFP may help distinguish gaze directed at a person (near) from gaze directed at the background behind them (far).

Are video-based eye trackers accurate enough to determine a BFP? To answer this question, we need to know how the error (inaccuracy) in the individual eye signals propagates to the error in the estimated BFP position. The relationship between two inaccurate gaze directions and the distance to the BFP is complex (see Fig. 1C in Hooge, Hessels, & Nyström, 2019). The estimated depth component of the BFP depends on vergence, the angle between the lines of sight of the left and right eyes (see Fig. 10). When the BFP is close to the observer, the vergence angle is large; as the BFP moves farther away, the vergence angle becomes smaller.

Figure 10 shows that a fixed gaze error (illustrated by the dashed lines) has a small impact on the estimated BFP distance when the fixation point is near, due to the steep vergence angle. In contrast, the same level of gaze error can lead to substantial errors in the estimated BFP distance when the fixation point lies far from the observer, where the vergence angle is shallow. Hooge et al. (2019) claimed that the currently available pupil-based eye trackers are poorly suited for estimating vergence and the distance to the BFP.

In short, using the BFP estimated with a pupil-based eye tracker to decide whether or not someone is looking inside a VOI may be problematic, especially for VOIs that are located at a greater distance from the observer. A potential solution is to enlarge the VOIs for objects and regions that are farther away from the observer.

There are also other potential solutions. The most straightforward is to use an eye-tracking method that is more accurate than the current state-of-the-art video-based eye trackers. The accuracy of pupil-based video eye trackers is, among other factors, limited by the pupil size artefact (PSA, Choe, Blake, & Lee, 2016; Drewes, Zhu, Hu, & Hu, 2014; Hooge et al., 2019; Hooge, Niehorster, Hessels, Cleveland, & Nyström, 2021, 2025a; Jaschinski, 2016; Wildenmann & Schaeffel, 2013; Wyatt, 2010). The PSA is a measurement error in video-based pupil–CR eye trackers, which rely on the pupil center to estimate gaze direction. When the pupil dilates or constricts, its centroid does not remain geometrically stable relative to the optical axis. Consequently, the estimated gaze position shifts, even though the eyeball itself does not move.

Calibration methods have been proposed to compensate for the PSA (e.g., Drewes et al., 2014). For example, Hooge et al. (2025a) demonstrated that the inaccuracy of an SR Research EyeLink 1000 Plus can be reduced by 50%. Whether this level of improvement is sufficient remains unclear, but it represents a significant step forward. An even better solution would be to adopt eye trackers that do not rely on the pupil to estimate gaze direction. Promising candidates include iris- and limbus-based eye trackers. The key question is whether affordable (wearable) eye trackers with sufficient accuracy will become available in the near future.

#### Do we need explicit AOIs?

With the advent of increased computing power and advances in image processing and object recognition tools, some authors (e.g., Alam & Jianu, 2016; Jianu & Alam, 2017) argue that AOIs may become obsolete in the future. They view the analysis of eye movements purely within recorded spatial coordinates as restrictive and propose replacing AOIs with more flexible constructs such as DOIs^13^ (Data-of-Interest). Another approach aiming to circumvent the need for predefined AOIs is Deep-SAGA (Deane et al., 2023), which automatically annotates all objects in an image or video, and uses the labels to determine which objects are looked at.

Proponents of these developments are optimistic, as such automated approaches have the potential to simplify and accelerate workflows in eye-tracking research. However, we believe that the shift to full automation is not without its drawbacks. If the entire process of annotating gaze data is handled by software, many critical decisions, including those discussed in the current article (see the “Deciding on the AOI” section), will be made implicitly by the underlying algorithms. These decisions are often closely tied to the research question, the task participants perform, and the quality of the eye-tracking data (see also the “About segmentation of whole scenes and movies” section).

We contend that before automated annotation methods can be broadly adopted in eye tracking research, they require thorough validation (e.g., see the list in “About segmentation of whole scenes and movies” section). Yet the question remains: how should this validation be conducted?

#### Validation of automated segmentation and annotation methods

How can automatic methods for segmentation (for AOI production) and annotation be validated? In this section, we discuss two approaches, without claiming to be exhaustive. The examples are intended as inspiration for researchers who wish to validate automatic methods.

The first validation approach concerns a complete data analysis pipeline for gaze-to-face mapping. Such a pipeline could, for example, provide automatic fixation annotation for gaze directed at faces. In a pilot experiment, a researcher interested in parent–child interaction could instruct the child to look several times at the father’s left eye, then the right eye, the nose, and then the mouth. The same procedure could be repeated for the parent, who may additionally be instructed to look next to the head or at the body. These instructions could be delivered via an audio file and earphones.

The pilot could include data from a diverse set of parents and children, for instance, from different ethnic backgrounds, to help rule out potential biases in the automatic method. This ensures that the researcher knows exactly what the data analysis should yield. If the automatic method’s output does not match the specified behavior, the validation is considered unsuccessful.

Validation, however, can extend beyond this. For example, Hessels, Niehorster, Kemner, and Hooge (2017) tested their automatic fixation detector on eye-tracking data of very poor quality (i.e., with high imprecision and substantial data loss). A similar procedure could be applied to the empirical validation described above. The test could be repeated under conditions where participants move extensively or where lighting is suboptimal. This would allow researchers to determine under which representative circumstances the automatic method still performs reliably. As an additional approach, eye-tracking data could also be artificially degraded by adding noise (e.g., as in Holmqvist et al. (2012) and Hessels et al. (2017)). Other forms of degradation may consist of lowering the contrast of or adding noise to the scene camera’s images.

In the second method, we focus on validating automatic AOI production using SAM 2 (Ravi et al., 2024). SAM 2 is capable of segmenting objects and generating a mask that fits them. To convert this mask into an AOI, however, a margin must be added around it that is at least as wide as the expected inaccuracy (see “Should the AOIs be small or large?” section). This margin can, for instance, be implemented in OpenCV using the morphological operation *dilate*.

Consider, for example, the question of how many viewers look at the horse Black Beauty in the children’s television series of the same name from the 1970s. As demonstration material, we can take a short clip (The Adventures of Black Beauty Opening Theme 1972) and let SAM 2 segment Black Beauty (the black horse) in every frame where it is present. The central question then becomes: how can we validate the AOIs generated with SAM 2 and OpenCV?

One approach is to have human annotators evaluate the quality of the AOIs. Yet the suitability of human annotators for this task is debatable. Hooge, Niehorster, Nyström, Andersson, and Hessels (2018) point out that untrained annotators may apply inconsistent internal criteria, that their criteria may shift over time, and that their limited working memory constrains how many rules they can apply consistently. Some of these issues can be mitigated through training and the establishment of a standardized evaluation procedure. Such procedures work well for short clips, but entire episodes of Black Beauty require much more effort. For large collections of material, human annotators might instead evaluate a random sample.

Another way to test the automatic method is to create controlled test films or images designed by the researcher, for example, with horses walking behind cars or fences, in the dark, among other horses or zebra’s, etc. The test material should be representative of the conditions of the stimulus material.

A further option is to compare AOIs generated by different automatic methods. Parameters such as AOI area, AOI centroid position, and whether an AOI is detected at all can then be tracked across film frames and compared between methods. Different outcomes across methods are a strong indication to inspect what is happening within the respective algorithms.

It is important to note that not every individual researcher needs to validate automatic annotation or segmentation algorithms independently. Often, time itself serves as a filter: when new algorithms are introduced, whether for segmentation or for related tasks such as saccade and fixation detection, it takes time for the research community to establish their reliability and suitability. For example, the SR Research saccade detector was once new, but has since become a widely accepted standard. This observation is not a call to suspend critical judgment of new methods, but rather a reminder that validation is a gradual process carried out collectively by the field.

### Common issues with AOIs

#### AOI analysis based on fixations or gaze samples?

This question has also been addressed by Holmqvist et al. (2011, p. 224). They highlight the differences in outcomes depending on which unit of analysis is chosen. Using gaze samples may give higher resolution and capture all gaze points, while using fixations provides cleaner, event-based data (e.g., no inclusion of saccades, and blinks, periods without clear visual perception). We want to add that an advantage of the fixation-based approach is that the AOI analysis is less sensitive to imprecision in the eye-tracking data. Holmqvist et al. (2011, p. 224) warn that inconsistent use of fixation- or sample-based methods across studies can lead to incomparable results. Researchers are urged to be explicit about their choice and to consider how that choice affects the validity and interpretability of their findings. We would like to add guidance on when researchers should use gaze-sample-based versus fixation-based analyses.

An AOI analysis using individual gaze samples can always be performed. In contrast, fixation-based AOI analysis requires reliable fixation classification, which is only possible when the imprecision of the eye-tracking data is not extremely high (Hessels et al., 2016b) and the eye tracker’s sampling rate is sufficiently high. An advantage of the fixation-based approach is that the AOI analysis is more robust to imprecision in the eye-tracking data, as each fixation aggregates multiple samples. Another example is noted by Friedrich et al. (2017), who state: “Due to the problem of the clear distinction between fixation, saccades, and smooth pursuits in dynamic task environments, the data analysis was based on the raw eye movement and ...”. Moreover, not all researchers have access to algorithms capable of performing accurate classification on their eye tracking data.

Another relevant point is that not everyone agrees on what exactly constitutes a fixation (Hessels, Niehorster, Nyström, Andersson, & Hooge, 2018b). This issue particularly arises when an observer needs to make a smooth pursuit eye movement in order to fixate a moving object. We will not go into this in detail here, but for anyone wishing to avoid this debate when conducting an AOI analysis, it is always safe to opt for a sample-based analysis instead.

In summary, we see no major disadvantages to either a fixation-based or a sample-based approach.

#### Ethical issues with AOI production

Do ethics play a role in the way AOIs are designed? At first sight, AOI production may appear to be a purely technical tool for structuring gaze data. Yet the act of defining AOIs is not necessarily neutral. It is an interpretative step that can determine which elements of a stimulus are emphasized and how results are ultimately interpreted.

This becomes especially significant in studies using, for example, real-world scenes, videos of eye tracker scene cameras, or social interactions. In these contexts, researchers typically face a choice: they can design AOIs by hand or rely on automated methods. Both approaches, however, bring their own ethical challenges.

Hand-drawn AOIs are vulnerable to subjectivity. Humans may consciously or unconsciously introduce bias when deciding where boundaries are placed and which details are included. Even small differences in how AOIs are drawn can change the data that are captured and, as a result, alter the conclusions of the study. Automated AOI methods, by contrast, are not free from bias either. Algorithms are built on assumptions, trained on particular datasets, and shaped by the goals of their developers. As a result, automated AOI production may reproduce hidden biases or introduce systematic distortions that are not immediately visible to researchers.

Because both human and algorithmic AOI design involve potential bias, it is difficult, if not impossible, to eliminate these risks. What researchers can do, however, is act with transparency. The best practice is to clearly document how AOIs were created, justify why a particular method was chosen, and be explicit about the limitations or potential biases inherent in those choices. Only by being transparent about the production and nature of AOIs can researchers allow others to critically evaluate the validity of the findings. As a result, automated AOI production may reproduce hidden biases or introduce systematic distortions that are not immediately visible to researchers (see e.g., Oyeniran, Adewusi, Adeleke, Akwawa, & Azubuko, 2022; Ravi et al., 2024, for how such biases may be addressed).

#### AOIs inspired by gaze data

In the introduction of this article (in the “The scope of this article” section), we stated that our primary interest lies in AOIs that are defined prior to inspecting the eye tracking data. In this section, we discuss whether and under what circumstances it may be desirable or useful to examine eye-tracking data before designing AOIs.

When AOI statistics are used to describe viewing behavior, it is entirely appropriate for researchers to first consult eye tracking data (e.g., in the form of a heat map or scanpaths) to understand where observers have looked. Such visualizations can guide the design of meaningful AOIs that effectively capture or summarize gaze behavior. However, when carrying out a confirmatory, hypothesis-testing experiment, the aim should always be to define AOIs in advance, before looking at the data.

In some cases, AOIs can be defined based on fixations from other observers or other parts of the study. For example, Foulsham and Kingstone (2013) tested whether participants “looked back” at details when looking at an image for a second time, by defining AOIs around each person’s fixations from encoding.

More generally, cases where it is appropriate to examine eye tracking data before deciding on the AOIs include:

- Assessing data quality: As described in “Deciding on the AOI” section, one factor that can influence the minimum size of an AOI is the inaccuracy of the eye tracking data. Inaccuracy determines how wide a margin around an object or region should be defined. The only way to make an informed decision about such margins is to examine representative data. That is data that reflects the expected quality of eye-tracking in the actual study.
- Investigating the conspicuity area of AOIs, which informs the appropriate AOI size for determining whether a participant has located the target.
- Justifying the use of large AOIs: In some contexts (such as sparse stimuli like human faces) very large AOIs may be appropriate. This may depend on how gaze is distributed, which in turn can be influenced by task instructions or participant background. For instance, individuals who have undergone eyelid surgery demonstrate different gaze patterns at faces (Cheng, Xia, Shi, & Zhang, 2025).
- Investigating the scale at which participants interact with the stimulus: are they looking at entire faces, or specifically at noses or ears? Are they looking at a stove or the burner controls of the stove (Fig. 7C).
- Making decisions about how to define AOIs in complex scenarios, such as overlapping objects or objects in close proximity.

We strongly recommend that researchers new to eye tracking spend time observing gaze data in detail to build an intuitive understanding of how their participants and eye tracker behave. Gaining this experience is essential for designing effective AOIs.

## Conclusion

We advise researchers to

- Pay enough attention to the decision process on AOIs (e.g., number, size, shape). They should at least take into account the level of detail concerning objects, the inaccuracy of the eye tracking data, the conspicuity of objects, the research question and the task for the participant.
- Report on AOI properties in their articles, especially when AOIs are hand-drawn. We would also like to encourage reviewers and editors to ensure that greater attention is given to the reporting of AOIs to enable reproduction.
- Consider transforming the eye tracking data to the frame of reference of the object(s) looked at when using data from wearable eye tracking glasses.
- Consider to learn how to program (Niehorster et al., 2025b, p. 19) as this facilitates the empirical approach in decision making that we advocated for earlier in this article and elsewhere (see Hessels et al., 2025b). Researchers who scripted their AOI production process and analysis, are much more flexible in decision-making concerning their AOIs and, for example, in motivating their AOIs in the review process, because they can relatively easily re-run with different parameters.

## Data Availability

Not applicable.

## Footnotes

1 or parts of video frames of dynamic stimuli or scene camera recordings of a wearable eye tracker

2 This point has been made earlier by Orquin and Holmqvist (2018) by stating that AOI dimensions should be clearly documented to enhance study replicability. It also aligns with the reporting guidelines of Dunn et al. (2024).

3 If the diameter of the AOI is small, a margin should be added to account for the inaccuracy of the eye tracker (see the “Should the AOIs be small or large?” section).

4 In the psychophysics literature the neighboring elements are usually referred to as flankers.

5 see https://github.com/ischtz/gridfix for a toolbox

6 SMI has been out of business since 2017, but we assume BeGaze is still used. GazeIntelligence, a company founded by former SMI employees, still supports SMI users.

7 Titta is available from https://github.com/marcus-nystrom/Titta (Python) and https://github.com/dcnieho/Titta (MATLAB).

8 This should be done using a non-compressed or losslessly compressed image format; we recommend PNG. JPG is a lossy format and thus should not be used.

9 An example of a distributed AOI in marketing research is a *brand* AOI (Pieters & Wedel, 2004), consisting of all Heineken logo’s from a Heineken advertisement.

10 See Webinar - Implementing text/reading experiments in SR Research Experiment Builder, https://www.youtube.com/watch?v=3oIrhaWUKvY.

11 Eye-tracking glasses can be equipped with a magnetic inertial measurement unit (MIMU) that measures angular velocities and linear accelerations. However, this does not solve the problem of the unknown relation between the head and the world. Transforming these MIMU signals into the orientation and position of the head is still prone to uncertainties (Hessels et al., 2022; Hooge, Niehorster, Nyström, & Hessels, 2024). Subsequently, combining head orientation and position signals with eye orientation signals to determine where in the world someone is looking is also not straightforward because the rotational axes of the eyes do not align with those of the head.

12 gazeMapper is compatible with a variety of wearable eye-tracking glasses, including SMI ETG 1, SMI ETG 2, Tobii Pro Glasses 2, Tobii Pro Glasses 3, AdHawk MindLink, Pupil Core, Pupil Invisible, Pupil Neon, and SeeTrue ST ONE.

13 also called gaze to object mapping (GTOM) which is well suited for computer-generated visual content (e.g., websites, virtual environments). Gaze coordinates can be automatically linked to the content of the visualization.

## Declarations

### Open Practices Statement

There are no data or code associated with the present article.

## References

[CR1] Akahori, W., Hirai, T., Kawamura, S., & Morishima, S. (2016). Region-of-interest-based subtitle placement using eye-tracking data of multiple viewers. In *Proceedings of the ACM international conference on interactive experiences for TV and online video* (pp. 123–128).

[CR2] Al-Azawi, M., Yang, Y., & Istance, H. (2014). A new gaze points agglomerative clustering algorithm and its application in regions of interest extraction. In *2014 IEEE International Advance Computing Conference (IACC)* (pp. 946–951). IEEE.

[CR3] Alam, S. S., & Jianu, R. (2016). Analyzing eye-tracking information in visualization and data space: From where on the screen to what on the screen. *IEEE Transactions on Visualization and Computer Graphics,* *23*(5), 1492–1505. 10.1109/TVCG.2016.2535340 26930687

[CR4] Alinaghi, N., Hollendonner, S., & Giannopoulos, I. (2024). MYFix: Automated fixation annotation of eye-tracking videos. *Sensors,* *24*(9), 2666. 10.3390/s24092666 PMC11085856 38732772

[CR5] Amati, M., Parmehr, E. G., McCarthy, C., & Sita, J. (2018). How eye-catching are natural features when walking through a park? Eye-tracking responses to videos of walks. *Urban Forestry & Urban Greening,* *31*, 67–78.

[CR6] Auyeung, B., Lombardo, M. V., Heinrichs, M., Chakrabarti, B., Sule, A., Deakin, J. B., Bethlehem, R., Dickens, L., Mooney, N., Sipple, J. A., et al. (2015). Oxytocin increases eye contact during a real-time, naturalistic social interaction in males with and without autism. *Translational Psychiatry,* *5*(2), e507. 10.1038/tp.2014.146 PMC4445747 25668435

[CR7] Ayala, N., Mardanbegi, D., Zafar, A., Niechwiej-Szwedo, E., Cao, S., Kearns, S., Irving, E., & Duchowski, A. T. (2024). Does fiducial marker visibility impact task performance and information processing in novice and low-time pilots? *Computers & Graphics,* *119*, 103889.

[CR8] Baltrušaitis, T., Robinson, P., & Morency, L. -P. (2016). OpenFace: An open source facial behavior analysis toolkit. In *2016 IEEE winter conference on applications of computer vision (WACV)* (pp. 1–10). IEEE.

[CR9] Batliner, M., Hess, S., Ehrlich-Adám, C., Lohmeyer, Q., & Meboldt, M. (2020). Automated areas of interest analysis for usability studies of tangible screen-based user interfaces using mobile eye tracking. *AI EDAM,* *34*(4), 505–514.

[CR10] Benjamins, J. S., Hessels, R. S., & Hooge, I. T. C. (2018). Gazecode: Open-source software for manual mapping of mobile eye-tracking data. In *Proceedings of the 2018 ACM symposium on eye tracking research & applications* (pp. 1–4).

[CR11] Bonikowski, L., Gruszczyński, D., & Matulewski, J. (2021). Open-source software for determining the dynamic areas of interest for eye tracking data analysis. *Procedia Computer Science,* *192*, 2568–2575.

[CR12] Borji, A., & Tanner, J. (2015). Reconciling saliency and object center-bias hypotheses in explaining free-viewing fixations. *IEEE Transactions on Neural Networks and Learning Systems,* *27*(6), 1214–1226. 10.1109/TNNLS.2015.2480683 26452292

[CR13] Bouma, H. (1970). Interaction effects in parafoveal letter recognition. *Nature,* *226*(5241), 177–178. 10.1038/226177a0 5437004

[CR14] Busjahn, T., & Tamm, S. (2021). A deeper analysis of AOI coverage in code reading. In *ACM symposium on eye tracking research and applications* (pp. 1–7).

[CR15] Bykowski, A., & Kupiński, S. (2018). Feature matching and ArUco markers application in mobile eye tracking studies. In *2018 signal processing: Algorithms, architectures, arrangements, and applications (SPA)* (pp. 255–260). IEEE.

[CR16] Callemein, T., Van Beeck, K., Brône, G., & Goedemé, T. (2019). Automated analysis of eye-tracker-based human-human interaction studies. In *Information science and applications 2018: ICISA 2018* (pp. 499–509). Springer.

[CR17] Cao, Z., Hidalgo, G., Simon, T., Wei, S.-E., & Sheikh, Y. (2019). Openpose: Realtime multi-person 2D pose estimation using part affinity fields. *IEEE Transactions on Pattern Analysis and Machine Intelligence,* *43*(1), 172–186. 10.1109/TPAMI.2019.2929257 31331883

[CR18] Cave, K. R., & Wolfe, J. M. (1990). Modeling the role of parallel processing in visual search. *Cognitive Psychology,* *22*(2), 225–271. 10.1016/0010-0285(90)90017-x 2331857

[CR19] Cheng, Z., Xia, W., Shi, J., & Zhang, C. (2025). Differences in gaze patterns for facial areas of the Asian human face between female patients undergoing upper blepharoplasty and nonoperators: An eye-tracking analysis. *Facial Plastic Surgery*. 10.1055/a-2589-3766 40239955

[CR20] Choe, K. W., Blake, R., & Lee, S.-H. (2016). Pupil size dynamics during fixation impact the accuracy and precision of video-based gaze estimation. *Vision Research,* *118*, 48–59. 10.1016/j.visres.2014.12.018 25578924

[CR21] Christ, S. E., & Abrams, R. A. (2006). Abrupt onsets cannot be ignored. *Psychonomic Bulletin & Review,* *13*, 875–880. 10.3758/bf03194012 17328388

[CR22] Clay, V., König, P., & Koenig, S. (2019). Eye tracking in virtual reality. *Journal of Eye Movement Research,* *12*(1), 3. 10.16910/jemr.12.1.3 PMC7903250 33828721

[CR23] Clayden, A. C., Fisher, R. B., & Nuthmann, A. (2020). On the relative (un) importance of foveal vision during letter search in naturalistic scenes. *Vision Research,* *177*, 41–55. 10.1016/j.visres.2020.07.005 32957035

[CR24] Cornelissen, T. H., & Võ, M.L.-H. (2017). Stuck on semantics: Processing of irrelevant object-scene inconsistencies modulates ongoing gaze behavior. *Attention, Perception, & Psychophysics,* *79*(1), 154–168. 10.3758/s13414-016-1203-7 27645215

[CR25] Culemann, W., & Heine, A. (2024). Eyeflow studio: An extensible gui-based tool for dynamic eye-tracking data processing and analysis. Poster presented at the European Conference on Eye Movements (ECEM 2024).

[CR26] Dawson, J., Kingstone, A., & Foulsham, T. (2021). Theory of mind affects the interpretation of another person’s focus of attention. *Scientific Reports,* *11*(1), 17147. 10.1038/s41598-021-96513-2 PMC8387431 34433836

[CR27] De La Hogue, T., Mouratille, D., Causse, M., & Imbert, J.-P. (2024). Argaze: An open and flexible software library for gaze analysis and interaction.

[CR28] Deane, O., Toth, E., & Yeo, S.-H. (2023). Deep-SAGA: A deep-learning-based system for automatic gaze annotation from eye-tracking data. *Behavior Research Methods,* *55*(3), 1372–1391. 10.3758/s13428-022-01833-4 PMC10126076 35650384

[CR29] Dik, V. K., Hooge, I. T. C., van Oijen, M. G. H., & Siersema, P. D. (2016). Measuring gaze patterns during colonoscopy: A useful tool to evaluate colon inspection? *European Journal of Gastroenterology & Hepatology,* *28*(12), 1400–1406. 10.1097/MEG.0000000000000717 27769078

[CR30] Drewes, J., Feder, S., & Einhäuser, W. (2021). Gaze during locomotion in virtual reality and the real world. *Frontiers in Neuroscience,* *15*, 656913. 10.3389/fnins.2021.656913 PMC8180583 34108857

[CR31] Drewes, J., Zhu, W., Hu, Y., & Hu, X. (2014). Smaller is better: Drift in gaze measurements due to pupil dynamics. *PloS ONE,* *9*(10), e111197. 10.1371/journal.pone.0111197 PMC4206464 25338168

[CR32] Drusch, G., Bastien, J., & Paris, S. (2014). Analysing eye-tracking data: From Scanpaths and heatmaps to the dynamic visualisation of areas of interest. *Advances in Science, Technology, Higher Education and Society in the Conceptual Age, STHESCA,* *20*(205), 25.

[CR33] Duchowski, A. T., Gehrer, N. A., Schönenberg, M., & Krejtz, K. (2019). Art facing science: Artistic heuristics for face detection: Tracking gaze when looking at faces. In *Proceedings of the 11th ACM symposium on eye tracking research & applications* (pp. 1–5).

[CR34] Dunn, M. J., Alexander, R. G., Amiebenomo, O. M., Arblaster, G., Atan, D., Erichsen, J. T., Ettinger, U., Giardini, M. E., Gilchrist, I. D., Hamilton, R., et al. (2024). Minimal reporting guideline for research involving eye tracking (2023 edition). *Behavior Research Methods,* *56*(5), 4351–4357. 10.3758/s13428-023-02187-1 PMC11225961 37507649

[CR35] Dupont, L., Antrop, M., & Van Eetvelde, V. (2014). Eye-tracking analysis in landscape perception research: Influence of photograph properties and landscape characteristics. *Landscape Research,* *39*(4), 417–432.

[CR36] Engel, F. L. (1971). Visual conspicuity, directed attention and retinal locus. *Vision Research,* *11*(6), 563–575. 10.1016/0042-6989(71)90077-0 5558576

[CR37] Engel, F. L. (1977). Visual conspicuity, visual search and fixation tendencies of the eye. *Vision Research,* *17*(1), 95–108. 10.1016/0042-6989(77)90207-3 855214

[CR38] Eraslan, S., Yesilada, Y., & Harper, S. (2020). “The best of both worlds!”: Integration of web page and eye tracking data driven approaches for automatic AOI detection. *ACM Transactions on the Web,* *14*(1), 1–31.

[CR39] Falck-Ytter, T. (2008). Face inversion effects in autism: A combined looking time and pupillometric study. *Autism Research,* *1*(5), 297–306. 10.1002/aur.45 19360681

[CR40] Feldman, J. (2003). What is a visual object? *Trends in Cognitive Sciences,* *7*(6), 252–256. 10.1016/s1364-6613(03)00111-6 12804691

[CR41] Fichtel, E., Lau, N., Park, J., Henrickson Parker, S., Ponnala, S., Fitzgibbons, S., & Safford, S. D. (2019). Eye tracking in surgical education: gaze-based dynamic area of interest can discriminate adverse events and expertise. *Surgical Endoscopy,* *33*, 2249–2256. 10.1007/s00464-018-6513-5 30341656

[CR42] Foulsham, T., & Kingstone, A. (2013). Where have eye been? Observers can recognise their own fixations. *Perception,* *42*(10), 1085–1089. 10.1068/p7562 24494439

[CR43] Foulsham, T., & Kingstone, A. (2025). Covert orienting: The dark matter of social attention. *Trends in Cognitive Sciences,* *29*(7), 597–599. 10.1016/j.tics.2025.05.001 40410094

[CR44] Foulsham, T., & Underwood, G. (2008). What can saliency models predict about eye movements? Spatial and sequential aspects of fixations during encoding and recognition. *Journal of Vision,* *8*(2), 6. 10.1167/8.2.6 18318632

[CR45] Foulsham, T., Walker, E., & Kingstone, A. (2011). The where, what and when of gaze allocation in the lab and the natural environment. *Vision Research,* *51*(17), 1920–1931. 10.1016/j.visres.2011.07.002 21784095

[CR46] Foulsham, T., Wybrow, D., & Cohn, N. (2016). Reading without words: Eye movements in the comprehension of comic strips. *Applied Cognitive Psychology,**30*(4), 566–579.

[CR47] Fredericksen, R., Bex, P. J., & Verstraten, F. A. (1997). How big is a Gabor patch, and why should we care? *Journal of the Optical Society of America A,* *14*(1), 1–12. 10.1364/josaa.14.000001 8988615

[CR48] Friedrich, M., Rußwinkel, N., & Möhlenbrink, C. (2017). A guideline for integrating dynamic areas of interests in existing set-up for capturing eye movement: Looking at moving aircraft. *Behavior Research Methods,* *49*, 822–834. 10.3758/s13428-016-0745-x 27287446

[CR49] Fuhl, W., Kübler, T. C., Santini, T., & Kasneci, E. (2018). Automatic generation of saliency-based areas of interest for the visualization and analysis of eye-tracking data. In *VMV* (pp. 47–54).

[CR50] Galfano, G., Dalmaso, M., Marzoli, D., Pavan, G., Coricelli, C., & Castelli, L. (2012). Eye gaze cannot be ignored (but neither can arrows). *Quarterly Journal of Experimental Psychology,* *65*(10), 1895–1910. 10.1080/17470218.2012.663765 22512343

[CR51] Garrido-Jurado, S., Muñoz-Salinas, R., Madrid-Cuevas, F. J., & Marín-Jiménez, M. J. (2014). Automatic generation and detection of highly reliable fiducial markers under occlusion. *Pattern Recognition,* *47*(6), 2280–2292.

[CR52] Gehrer, N. A., Svaldi, J., & Duchowski, A. (2024). Assessment of body-related attention processes via mobile eye tracking: A pilot study to validate an automated analysis pipeline. In *Proceedings of the 2024 symposium on eye tracking research and applications* (pp. 1–7).

[CR53] Gidlöf, K., Wallin, A., Dewhurst, R., & Holmqvist, K. (2013). Using eye tracking to trace a cognitive process: Gaze behaviour during decision making in a natural environment. *Journal of Eye Movement Research,* *6*(1), 3.

[CR54] Gilchrist, I. D., & Harvey, M. (2000). Refixation frequency and memory mechanisms in visual search. *Current Biology,* *10*(19), 1209–1212. 10.1016/s0960-9822(00)00729-6 11050390

[CR55] Glaholt, M. G., Wu, M., & Reingold, E. M. (2009). Predicting preference from fixations. *PsychNology J.,* *7*(2), 141–158.

[CR56] Godfroid, A. (2019). *Eye tracking in second language acquisition and bilingualism: A research synthesis and methodological guide*. Routledge.

[CR57] Gullberg, M., & Holmqvist, K. (2006). What speakers do and what addressees look at: Visual attention to gestures in human interaction live and on video. *Pragmatics & Cognition,* *14*(1), 53–82.

[CR58] Hayes, T. R., & Henderson, J. M. (2021). Looking for semantic similarity: What a vector-space model of semantics can tell us about attention in real-world scenes. *Psychological Science,* *32*(8), 1262–1270. 10.1177/0956797621994768 PMC8726595 34252325

[CR59] Henderson, J. M., & Hollingworth, A. (1999). High-level scene perception. *Annual Review of Psychology,* *50*(1), 243–271. 10.1146/annurev.psych.50.1.243 10074679

[CR60] Hermens, F. (2024). Automatic object detection for behavioural research using YOLOv8. *Behavior Research Methods,* *56*(7), 7307–7330. 10.3758/s13428-024-02420-5 PMC11362367 38750389

[CR61] Hessels, R. S., Hooge, I. T. C., & Kemner, C. (2016a). An in-depth look at saccadic search in infancy. *Journal of Vision,* *16*(8), 10. 10.1167/16.8.10 27299770

[CR62] Hessels, R. S., Kemner, C., van den Boomen, C., & Hooge, I. T. C. (2016b). The area-of-interest problem in eyetracking research: A noise-robust solution for face and sparse stimuli. *Behavior Research Methods,* *48*, 1694–1712. 10.3758/s13428-015-0676-y PMC5101255 26563395

[CR63] Hessels, R. S., Niehorster, D. C., Kemner, C., & Hooge, I. (2017). Noise-robust fixation detection in eye movement data: Identification by two-means clustering (I2MC). *Behavior Research Methods,* *49*(5), 1802–1823. 10.3758/s13428-016-0822-1 PMC5628191 27800582

[CR64] Hessels, R. S., Benjamins, J. S., Cornelissen, T. H., & Hooge, I. T. C. (2018a). A validation of automatically-generated areas-of-interest in videos of a face for eye-tracking research. *Frontiers in Psychology,* *9*, 1367. 10.3389/fpsyg.2018.01367 PMC6085555 30123168

[CR65] Hessels, R. S., Niehorster, D. C., Nyström, M., Andersson, R., & Hooge, I. T. C. (2018b). Is the eye-movement field confused about fixations and saccades? A survey among 124 researchers. Royal Society Open. *Science,* *5*(8), 180502. 10.1098/rsos.180502 PMC6124022 30225041

[CR66] Hessels, R. S., Benjamins, J. S., Van Doorn, A. J., Koenderink, J. J., Holleman, G. A., & Hooge, I. T. C. (2020). Looking behavior and potential human interactions during locomotion. *Journal of Vision,* *20*(10), 5. 10.1167/jov.20.10.5 PMC7545070 33007079

[CR67] Hessels, R. S., Benjamins, J. S., Niehorster, D. C., van Doorn, A. J., Koenderink, J. J., Holleman, G. A., de Kloe, Y. J., Valtakari, N. V., van Hal, S., & Hooge, I. T. C. (2022). Eye contact avoidance in crowds: A large wearable eye-tracking study. *Attention, Perception, & Psychophysics,* *84*(8), 2623–2640. 10.3758/s13414-022-02541-z PMC9630249 35996058

[CR68] Hessels, R. S., Teunisse, M. K., Niehorster, D. C., Nyström, M., Benjamins, J. S., Senju, A., & Hooge, I. T. C. (2023). Task-related gaze behaviour in face-to-face dyadic collaboration: Toward an interactive theory? *Visual Cognition,* *31*(4), 291–313.

[CR69] Hessels, R. S., Iwabuchi, T., Niehorster, D. C., Funawatari, R., Benjamins, J. S., Kawakami, S., Nyström, M., Suda, M., Hooge, I. T. C., Sumiya, M., Heijnen, J. I. P., Teunisse, M. K., & Senju, A. (2025a). Gaze behavior in face-to-face interaction: A cross-cultural investigation between Japan and the Netherlands. *Cognition,* *263*, 106174. 10.1016/j.cognition.2025.106174 40424776

[CR70] Hessels, R. S., Niehorster, D.C., Nyström, M., Andersson, R., Holleman, G. A., & Hooge, I. T. C. (2025b). The fundamentals of eye tracking part 5: The importance of piloting. *Behavior Research Methods,* *57*(8), 216. 10.3758/s13428-025-02737-9 PMC12222426 40603646

[CR71] Hessels, R. S., Nuthmann, A., Nyström, M., Andersson, R., Niehorster, D. C., & Hooge, I. T. C. (2025c). The fundamentals of eye tracking part 1: The link between theory and research question. *Behavior Research Methods,* *57*(1), 16. 10.3758/s13428-024-02544-8 PMC11638287 39668288

[CR72] Hofbauer, M., Kuhn, C. B., Püttner, L., Petrovic, G., & Steinbach, E. (2020). Measuring driver situation awareness using region-of-interest prediction and eye tracking. In *2020 IEEE International Symposium on Multimedia (ISM)* (pp. 91–95). IEEE.

[CR73] Hoffmann, A., Schiestl, S., Sinske, P., Gondan, M., Sachse, P., & Maran, T. (2024). Sharing and receiving eye-contact predicts mate choice after a 5-minute conversation: Evidence from a speed-dating study. *Archives of Sexual Behavior,* *53*(3), 959–968. 10.1007/s10508-023-02806-0 PMC10920202 38379110

[CR74] Holleman, G. A., Hessels, R. S., Kemner, C., & Hooge, I. T. C. (2020). Implying social interaction and its influence on gaze behavior to the eyes. *PLoS ONE,* *15*(2), e0229203. 10.1371/journal.pone.0229203 PMC7039466 32092089

[CR75] Holleman, G. A., Hooge, I. T. C., Huijding, J., Deković, M., Kemner, C., & Hessels, R. S. (2021). Gaze and speech behavior in parent–child interactions: The role of conflict and cooperation. *Current Psychology*, pp. 1–22.

[CR76] Holmqvist, K., Nyström, M., Andersson, R., Dewhurst, R., Jarodzka, H., & van de Weijer, J. (2011). *Eye tracking: A comprehensive guide to methods and measures*. Oxford: Oxford University Press.

[CR77] Holmqvist, K., Nyström, M., & Mulvey, F. (2012). Eye tracker data quality: What it is and how to measure it. In *Proceedings of the symposium on eye tracking research and applications* (pp. 45–52).

[CR78] Hooge, I. T. C., & Erkelens, C. J. (1996). Control of fixation duration in a simple search task. *Perception & Psychophysics,* *58*(7), 969–976. 10.3758/bf03206825 8920834

[CR79] Hooge, I. T. C., & Erkelens, C. J. (1999). Peripheral vision and oculomotor control during visual search. *Vision Research,* *39*(8), 1567–1575. 10.1016/s0042-6989(98)00213-2 10343822

[CR80] Hooge, I. T. C., Hessels, R. S., Niehorster, D. C., Andersson, R., Skrok, M. K., Konklewski, R., Stremplewski, P., Nowakowski, M., Tamborski, S., Szkulmowska, A., et al. (2025a). Eye tracker calibration: How well can humans refixate a target? *Behavior Research Methods,* *57*(1), 23. 10.3758/s13428-024-02564-4 PMC11659352 39702537

[CR81] Hooge, I. T. C., Hessels, R. S., & Nyström, M. (2019). Do pupil-based binocular video eye trackers reliably measure vergence? *Vision Research,* *156*, 1–9. 10.1016/j.visres.2019.01.004 30641092

[CR82] Hooge, I. T. C., Niehorster, D. C., Hessels, R. S., Cleveland, D., & Nyström, M. (2021). The pupil-size artefact (PSA) across time, viewing direction, and different eye trackers. *Behavior Research Methods*, pp. 1–21. 10.3758/s13428-020-01512-2 PMC8516786 33709298

[CR83] Hooge, I. T. C., Niehorster, D. C., Nyström, M., Andersson, R., & Hessels, R. S. (2018). Is human classification by experienced untrained observers a gold standard in fixation detection? *Behavior Research Methods,* *50*, 1864–1881. 10.3758/s13428-017-0955-x PMC7875941 29052166

[CR84] Hooge, I. T. C., Niehorster, D. C., Nyström, M., & Hessels, R. S. (2024). Large eye-head gaze shifts measured with a wearable eye tracker and an industrial camera. *Behavior Research Methods,* *56*(6), 5820–5833. 10.3758/s13428-023-02316-w PMC11335818 38200239

[CR85] Hooge, I. T. C., Nuthmann, A., Nyström, M., Niehorster, D. C., Holleman, G. A., Andersson, R., & Hessels, R. S. (2025b). The fundamentals of eye tracking part 2: From research question to operationalization. *Behavior Research Methods,* *57*(2), 73. 10.3758/s13428-024-02590-2 PMC11761893 39856471

[CR86] Hudson, R. (2023). Explicating exact versus conceptual replication. *Erkenntnis,* *88*(6), 2493–2514. 10.1007/s10670-021-00464-z PMC10300171 37388139

[CR87] Jakobi, D. N., Kern, T., Reich, D. R., Haller, P., & Jäger, L. A. (2025). PoTeC: A German naturalistic eye-tracking-while-reading corpus. *Behavior Research Methods,* *57*(8), 211. 10.3758/s13428-024-02536-8 PMC12208991 40588653

[CR88] Janik, S. W., Wellens, A. R., Goldberg, M. L., & Dell’Osso, L. F. (1978). Eyes as the center of focus in the visual examination of human faces. *Perceptual and Motor Skills,* *47*(3), 857–858. 10.2466/pms.1978.47.3.857 740480

[CR89] Jaschinski, W. (2016). Pupil size affects measures of eye position in video eye tracking: Implications for recording vergence accuracy. *Journal of Eye Movement Research,* *9*(4), 2.

[CR90] Jiang, S., Chen, W., & Kang, Y. (2021). Correlation evaluation of pilots’ situation awareness in bridge simulations via eye-tracking technology. *Computational Intelligence and Neuroscience,* *2021*(1), 15. 10.1155/2021/7122437 PMC8664503 34899896

[CR91] Jianu, R., & Alam, S. S. (2017). A data model and task space for data of interest (DOI) eye-tracking analyses. *IEEE Transactions on Visualization and Computer Graphics,* *24*(3), 1232–1245. 10.1109/TVCG.2017.2665498 28186899

[CR92] Jones, W., & Klin, A. (2013). Attention to eyes is present but in decline in 2-6-month-old infants later diagnosed with autism. *Nature,* *504*(7480), 427–431. 10.1038/nature12715 PMC4035120 24196715

[CR93] Just, M. A., & Carpenter, P. A. (1976). Eye fixations and cognitive processes. *Cognitive Psychology,* *8*(4), 441–480.

[CR94] Kang, Z., Mandal, S., Crutchfield, J., Millan, A., & McClung, S. N. (2016). Designs and algorithms to map eye tracking data with dynamic multielement moving objects. *Computational Intelligence and Neuroscience,* *2016*(1), 9354760. 10.1155/2016/9354760 PMC5048099 27725830

[CR95] Kirillov, A., Mintun, E., Ravi, N., Mao, H., Rolland, C., Gustafson, L., ... & Girshick, R. (2023). Segment anything. In *Proceedings of the IEEE/CVF international conference on computer vision*

[CR96] Kliegl, R., Nuthmann, A., & Engbert, R. (2006). Tracking the mind during reading: The influence of past, present, and future words on fixation durations. *Journal of Experimental Psychology: General,* *135*(1), 12–35. 10.1037/0096-3445.135.1.12 16478314

[CR97] Knoop-van Campen, C. A. N., Ter Doest, D., Verhoeven, L., & Segers, E. (2022). The effect of audio-support on strategy, time, and performance on reading comprehension in secondary school students with dyslexia. *Annals of Dyslexia*, pp 1–20. 10.1007/s11881-021-00246-w PMC9187546 34797513

[CR98] Kooi, F. L., Toet, A., Tripathy, S. P., & Levi, D. M. (1994). The effect of similarity and duration on spatial interaction in peripheral vision. *Spatial Vision,* *8*(2), 255–280. 10.1163/156856894x00350 7993878

[CR99] Kopácsi, L., Barz, M., Bhatti, O. S., & Sonntag, D. (2023). IMETA: An interactive mobile eye tracking annotation method for semi-automatic fixation-to-AOI mapping. In *Companion proceedings of the 28th international conference on intelligent user interfaces* (pp. 33–36).

[CR100] Körner, C., & Gilchrist, I. D. (2008). Memory processes in multiple-target visual search. *Psychological Research,* *72*, 99–105. 10.1007/s00426-006-0075-1 17021837

[CR101] Körner, H. M., Faul, F., & Nuthmann, A. (2023). Revisiting the role of attention in the “weapon focus effect”: Do weapons draw gaze away from the perpetrator under naturalistic viewing conditions? *Attention, Perception, & Psychophysics,* *85*(6), 1868–1887. 10.3758/s13414-022-02643-8 PMC10545598 36725782

[CR102] Körner, H. M., Faul, F., & Nuthmann, A. (2024). Is a knife the same as a plunger? Comparing the attentional effects of weapons and non-threatening unusual objects in dynamic scenes. *Cognitive Research: Principles and Implications,* *9*(1), 66. 10.1186/s41235-024-00579-1 PMC11461415 39379777

[CR103] Kübler, T. C., Sippel, K., Fuhl, W., Schievelbein, G., Aufreiter, J., Rosenberg, R., Rosenstiel, W., & Kasneci, E. (2015). Analysis of eye movements with eyetrace. In *Biomedical engineering systems and technologies: 8th international joint conference, BIOSTEC 2015, Lisbon, Portugal, January 12-15, 2015, Revised Selected Papers 8* (pp. 458–471). Springer.

[CR104] Lagmay, E. A. D., Rodrigo, M. M. T., et al. (2022). Enhanced automatic areas of interest (AOI) bounding boxes estimation algorithm for dynamic eye-tracking stimuli. *APSIPA Transactions on Signal and Information Processing, 11*(1).

[CR105] Laidlaw, K. E., Risko, E. F., & Kingstone, A. (2012). A new look at social attention: Orienting to the eyes is not (entirely) under volitional control. *Journal of Experimental Psychology: Human Perception and Performance,* *38*(5), 1132–1143. 10.1037/a0027075 22686696

[CR106] Le, D. D., Nguyen, T. K. C., Le, T. H., Nguyen, T. C. H., & Ngo, T. D. (2023). An eye tracking-based system for capturing visual strategies in reading of children with dyslexia. In *Proceedings of the 12th international symposium on information and communication technology* (pp. 856–862).

[CR107] Levi, D. M. (2008). Crowding-an essential bottleneck for object recognition: A mini-review. *Vision Research,* *48*(5), 635–654. 10.1016/j.visres.2007.12.009 PMC2268888 18226828

[CR108] Li, L.-J., Socher, R., & Fei-Fei, L. (2009). Towards total scene understanding: Classification, annotation and segmentation in an automatic framework. In *2009 IEEE conference on computer vision and pattern recognition* (pp. 2036–2043). IEEE.

[CR109] Liversedge, S. P., Olkoniemi, H., Zang, C., Li, X., Yan, G., Bai, X., & Hyönä, J. (2024). Universality in eye movements and reading: A replication with increased power. *Cognition,* *242*, 105636. 10.1016/j.cognition.2023.105636 37857054

[CR110] Malcolm, G. L., & Henderson, J. M. (2010). Combining top-down processes to guide eye movements during real-world scene search. *Journal of Vision,* *10*(2), 4. 10.1167/10.2.4 20462305

[CR111] Malcolm, G. L., Nuthmann, A., & Schyns, P. G. (2014). Beyond gist: Strategic and incremental information accumulation for scene categorization. *Psychological Science,* *25*(5), 1087–1097. 10.1177/0956797614522816 PMC4232276 24604146

[CR112] Maran, T., Hoffmann, A., & Sachse, P. (2022). Early lifetime experience of urban living predicts social attention in real world crowds. *Cognition,* *225*, 105099. 10.1016/j.cognition.2022.105099 35334252

[CR113] McConkie, G. W., Kerr, P. W., Reddix, M. D., & Zola, D. (1988). Eye movement control during reading: I. The location of initial eye fixations on words. *Vision Research,* *28*(10), 1107–1118. 10.1016/0042-6989(88)90137-x 3257013

[CR114] Mercier, J., Ertz, O., & Bocher, E. (2024). Quantifying dwell time with location-based augmented reality: Dynamic AOI analysis on mobile eye tracking data with vision transformer. *Journal of Eye Movement Research,* *17*(3), 3. 10.16910/jemr.17.3.3 PMC11165940 38863891

[CR115] Meyer, L., Josefsson, B., Vrotsou, K., Westin, C., & Lundberg, J. (2021). Evaluation of an AOI mapping and analysis tool for the identification of visual scan pattern. In *2021 IEEE/AIAA 40th Digital Avionics Systems Conference (DASC)* (pp. 1–8). IEEE.

[CR116] Müller, D., & Mann, D. (2021). Algorithmic gaze classification for mobile eye-tracking. In *ACM symposium on eye tracking research and applications* (pp. 1–4).

[CR117] Najemnik, J., & Geisler, W. S. (2005). Optimal eye movement strategies in visual search. *Nature,* *434*(7031), 387–391. 10.1038/nature03390 15772663

[CR118] Nguyen, D., Vrzakova, H., & Bednarik, R. (2016). WTP: Web-tracking plugin for real-time automatic AOI annotations. In *Proceedings of the 2016 ACM international joint conference on pervasive and ubiquitous computing: Adjunct* (pp. 1696–1705).

[CR119] Niehorster, D. C., Andersson, R., & Nyström, M. (2020). Titta: A toolbox for creating PsychToolbox and Psychopy experiments with Tobii eye trackers. *Behavior Research Methods,* *52*, 1970–1979. 10.3758/s13428-020-01358-8 PMC7575480 32128697

[CR120] Niehorster, D. C., Hessels, R. S., Nyström, M., Benjamins, J. S., & Hooge, I. T. C. (2025a). gazeMapper: A tool for automated world-based analysis of gaze data from one or multiple wearable eye trackers. *Behavior Research Methods,* *57*(7), 188. 10.3758/s13428-025-02704-4 PMC12134025 40461911

[CR121] Niehorster, D. C., Nyström, M., Hessels, R. S., Andersson, R., Benjamins, J. S., Hansen, D. W., & Hooge, I. T. C. (2025b). The fundamentals of eye tracking part 4: Tools for conducting an eye tracking study. *Behavior Research Methods,* *57*(1), 46. 10.3758/s13428-024-02529-7 PMC11703944 39762687

[CR122] Nuthmann, A. (2013). Not fixating at the line of text comes at a cost. *Attention, Perception, & Psychophysics,* *75*(8), 1604–1609. 10.3758/s13414-013-0581-3 24189941

[CR123] Nuthmann, A., & Clark, C. N. (2023). Pseudoneglect during object search in naturalistic scenes. *Experimental Brain Research,* *241*(9), 2345–2360. 10.1007/s00221-023-06679-6 PMC10471692 37610677

[CR124] Nuthmann, A., De Groot, F., Huettig, F., & Olivers, C. N. (2019). Extrafoveal attentional capture by object semantics. *PLoS ONE,* *14*(5), e0217051. 10.1371/journal.pone.0217051 PMC6532879 31120948

[CR125] Nuthmann, A., Engbert, R., & Kliegl, R. (2005). Mislocated fixations during reading and the inverted optimal viewing position effect. *Vision Research,* *45*(17), 2201–2217. 10.1016/j.visres.2005.02.014 15924936

[CR126] Nuthmann, A., & Henderson, J. M. (2010). Object-based attentional selection in scene viewing. *Journal of Vision,* *10*(8), 20. 10.1167/10.8.20 20884595

[CR127] Nuthmann, A., & Henderson, J. M. (2012). Using CRISP to model global characteristics of fixation durations in scene viewing and reading with a common mechanism. *Visual Cognition,* *20*(4–5), 457–494.

[CR128] Nuthmann, A., Schütz, I., & Einhäuser, W. (2020). Salience-based object prioritization during active viewing of naturalistic scenes in young and older adults. *Scientific Reports,* *10*(1), 22057. 10.1038/s41598-020-78203-7 PMC7745017 33328485

[CR129] Nuthmann, A., & Faul, F. (2026). The meaning of salience and the salience of meaning: Object prioritization within naturalistic scenes. PsyArXiv. 10.31234/osf.io/x53ce_v1

[CR130] Nyström, M., Hooge, I. T. C., Hessels, R. S., Andersson, R., Hansen, D. W., Johansson, R., & Niehorster, D. C. (2025). The fundamentals of eye tracking part 3: How to choose an eye tracker. *Behavior Research Methods,* *57*(2), 67. 10.3758/s13428-024-02587-x PMC11754381 39843609

[CR131] Oliva, A., & Torralba, A. (2006). Building the gist of a scene: The role of global image features in recognition. *Progress in Brain Research,* *155*, 23–36. 10.1016/S0079-6123(06)55002-2 17027377

[CR132] Orquin, J. L., Ashby, N. J., & Clarke, A. D. (2016). Areas of interest as a signal detection problem in behavioral eye-tracking research. *Journal of Behavioral Decision Making,* *29*(2–3), 103–115.

[CR133] Orquin, J. L., & Holmqvist, K. (2018). Threats to the validity of eye-movement research in psychology. *Behavior Research Methods,* *50*, 1645–1656. 10.3758/s13428-017-0998-z 29218588

[CR134] Osokin, D. (2018). Real-time 2D multi-person pose estimation on CPU: Lightweight openpose. arXiv:1811.12004.

[CR135] Otto, K., Castner, N., Geisler, D., & Kasneci, E. (2018). Development and evaluation of a gaze feedback system integrated into eyetrace. In *Proceedings of the 2018 ACM symposium on eye tracking research & applications* (pp. 1–5).

[CR136] Ouzts, A. D., Duchowski, A. T., Gomes, T., & Hurley, R. A. (2012). On the conspicuity of 3-D fiducial markers in 2-D projected environments. In *Proceedings of the symposium on eye tracking research and applications* (pp. 325–328).

[CR137] Over, E. A. B., Hooge, I. T. C., & Erkelens, C. J. (2006). A quantitative measure for the uniformity of fixation density: The Voronoi method. *Behavior Research Methods,* *38*, 251–261. 10.3758/bf03192777 16956102

[CR138] Oyeniran, C., Adewusi, A. O., Adeleke, A. G., Akwawa, L. A., & Azubuko, C. F. (2022). Ethical AI: Addressing bias in machine learning models and software applications. *Computer Science & IT Research Journal,* *3*(3), 115–126.

[CR139] Pajak, M., & Nuthmann, A. (2013). Object-based saccadic selection during scene perception: Evidence from viewing position effects. *Journal of Vision,* *13*(5), 2. 10.1167/13.5.2 23547104

[CR140] Papenmeier, F., & Huff, M. (2010). DynAOI: A tool for matching eye-movement data with dynamic areas of interest in animations and movies. *Behavior Research Methods,* *42*(1), 179–187. 10.3758/BRM.42.1.179 20160298

[CR141] Patching, G. R., & Jordan, T. R. (1998). Increasing the benefits of eye-tracking devices in divided visual field studies of cerebral asymmetry. *Behavior Research Methods, Instruments, & Computers,* *30*, 643–650.

[CR142] Paterson, K. B., & Jordan, T. R. (2010). Effects of increased letter spacing on word identification and eye guidance during reading. *Memory & Cognition,* *38*(4), 502–512. 10.3758/MC.38.4.502 20516230

[CR143] Pelli, D. G., & Tillman, K. A. (2008). The uncrowded window of object recognition. *Nature Neuroscience,* *11*(10), 1129–1135. 10.1038/nn.2187 PMC2772078 18828191

[CR144] Pfeiffer, T., & Renner, P. (2014). EyeSee3D: A low-cost approach for analyzing mobile 3D eye tracking data using computer vision and augmented reality technology. In *Proceedings of the symposium on eye tracking research and applications* (pp. 195–202).

[CR145] Pieters, R., & Wedel, M. (2004). Attention capture and transfer in advertising: Brand, pictorial, and text-size effects. *Journal of Marketing,* *68*(2), 36–50.

[CR146] Ravi, N., Gabeur, V., Hu, Y.-T., Hu, R., Ryali, C., Ma, T., Khedr, H., Rädle, R., Rolland, C., Gustafson, L., et al. (2024). SAM 2: Segment anything in images and videos. arXiv:2408.00714.

[CR147] Rayner, K., & Duffy, S. A. (1986). Lexical complexity and fixation times in reading: Effects of word frequency, verb complexity, and lexical ambiguity. *Memory & Cognition,* *14*(3), 191–201. 10.3758/bf03197692 3736392

[CR148] Richtsfeld, A., Mörwald, T., Prankl, J., Zillich, M., & Vincze, M. (2012). Segmentation of unknown objects in indoor environments. In *2012 IEEE/RSJ international conference on intelligent robots and systems* (pp. 4791–4796). IEEE.

[CR149] Rim, N. W., Choe, K. W., Scrivner, C., & Berman, M. G. (2021). Introducing point-of-interest as an alternative to area-of-interest for fixation duration analysis. *PLoS ONE,**16*(5), e0250170. 10.1371/journal.pone.0250170 PMC8109773 33970920

[CR150] Rogers, S. L., Speelman, C. P., Guidetti, O., & Longmuir, M. (2018). Using dual eye tracking to uncover personal gaze patterns during social interaction. *Scientific Reports,* *8*(1), 4271. 10.1038/s41598-018-22726-7 PMC5844880 29523822

[CR151] Rosenholtz, R. (2016). Capabilities and limitations of peripheral vision. *Annual Review of Vision Science,* *2*(1), 437–457. 10.1146/annurev-vision-082114-035733 28532349

[CR152] Rosenholtz, R. (2024). Visual attention in crisis. *Behavioral and Brain Sciences*, pp. 1–32. 10.1017/S0140525X24000323 38699816

[CR153] Russell, B. C., Torralba, A., Murphy, K. P., & Freeman, W. T. (2008). LabelMe: A database and web-based tool for image annotation. *International Journal of Computer Vision,* *77*, 157–173.

[CR154] Rutishauser, U., & Koch, C. (2007). Probabilistic modeling of eye movement data during conjunction search via feature-based attention. *Journal of Vision,* *7*(6), 5. 10.1167/7.6.5 17685788

[CR155] Sakano, N. (1963). The role of eye movements in various forms of perception. *Psychologia,* *6*(4), 215–227.

[CR156] Schotter, E. R., Berry, R. W., McKenzie, C. R. M., & Rayner, K. (2010). Gaze bias: Selective encoding and liking effects. *Visual Cognition,* *18*(8), 1113–1132.

[CR157] Shimojo, S., Simion, C., Shimojo, E., & Scheier, C. (2003). Gaze bias both reflects and influences preference. *Nature Neuroscience,* *6*(12), 1317–1322. 10.1038/nn1150 14608360

[CR158] Smith, R. (2007). An overview of the tesseract OCR engine. In *Ninth international conference on document analysis and recognition (ICDAR 2007)* (vol. 2, pp. 629–633). IEEE.

[CR159] Steichen, B. (2024). Computational methods to infer human factors for adaptation and personalization using eye tracking. In *A human-centered perspective of intelligent personalized environments and systems* (pp. 183–204). Springer.

[CR160] Stein, I., Jossberger, H., & Gruber, H. (2022). Investigating visual expertise in sculpture: A methodological approach using eye tracking. *Journal of Eye Movement Research,* *15*(2), 5. 10.16910/jemr.15.2.5 PMC9754618 36530479

[CR161] Sullivan, B., Ludwig, C. J., Damen, D., Mayol-Cuevas, W., & Gilchrist, I. D. (2021). Look-ahead fixations during visuomotor behavior: Evidence from assembling a camping tent. *Journal of Vision,* *21*(3), 13. 10.1167/jov.21.3.13 PMC7961111 33688920

[CR162] Tabuchi, M., & Hirotomi, T. (2022). Using fiducial marker for analyzing wearable eye-tracker gaze data measured while cooking. In *International Conference on Human-Computer Interaction* (pp. 192–204). Springer.

[CR163] Tang, S., Reilly, R. G., & Vorstius, C. (2012). EyeMap: A software system for visualizing and analyzing eye movement data in reading. *Behavior Research Methods,* *44*, 420–438. 10.3758/s13428-011-0156-y 21994183

[CR164] Tatler, B. W., Wade, N. J., Kwan, H., Findlay, J. M., & Velichkovsky, B. M. (2010). Yarbus, eye movements, and vision. *i-Perception*, *1*(1):7–27. 10.1068/i0382 PMC3563050 23396904

[CR165] Theeuwes, J. (2010). Top-down and bottom-up control of visual selection. *Acta Psychologica,* *135*(2), 77–99. 10.1016/j.actpsy.2010.02.006 20507828

[CR166] Toet, A., & Levi, D. M. (1992). The two-dimensional shape of spatial interaction zones in the parafovea. *Vision Research,* *32*(7), 1349–1357. 10.1016/0042-6989(92)90227-a 1455707

[CR167] Trefzger, M., Blascheck, T., Raschke, M., Hausmann, S., & Schlegel, T. (2018). A visual comparison of gaze behavior from pedestrians and cyclists. In *Proceedings of the 2018 ACM symposium on eye tracking research & applications* (pp. 1–5).

[CR168] Trukenbrod, H. A., & Engbert, R. (2007). Oculomotor control in a sequential search task. *Vision Research,* *47*(18), 2426–2443. 10.1016/j.visres.2007.05.010 17662332

[CR169] Tzamaras, H. M., Wu, H.-L., Moore, J. Z., & Miller, S. R. (2023). Shifting perspectives: A proposed framework for analyzing head-mounted eye-tracking data with dynamic areas of interest and dynamic scenes. In *Proceedings of the human factors and ergonomics society annual meeting* (Vol. 67(1), pp. 953–958). Los Angeles, CA: SAGE Publications Sage CA. 10.1177/21695067231192929 PMC10914345 38450120

[CR170] van der Laan, L. N., Hooge, I. T. C., de Ridder, D. T. D., Viergever, M. A., & Smeets, P. A. M. (2015). Do you like what you see? The role of first fixation and total fixation duration in consumer choice. *Food Quality and Preference,* *39*, 46–55.

[CR171] Vehlen, A., Standard, W., & Domes, G. (2022). How to choose the size of facial areas of interest in interactive eye tracking. *PLoS ONE,* *17*(2), e0263594. 10.1371/journal.pone.0263594 PMC8815978 35120188

[CR172] Viktorsson, C., Valtakari, N. V., Falck-Ytter, T., Hooge, I. T. C., Rudling, M., & Hessels, R. S. (2023). Stable eye versus mouth preference in a live speech-processing task. *Scientific Reports,* *13*(1), 12878. 10.1038/s41598-023-40017-8 PMC10409748 37553414

[CR173] Viviani, P. (1990). Eye movements in visual search: Cognitive, perceptual and motor control aspects. *Eye movements and their role in visual and cognitive processes*, pp. 353–393. 7492533

[CR174] Vlaskamp, B. N. S., & Hooge, I. T. C. (2006). Crowding degrades saccadic search performance. *Vision Research,* *46*(3), 417–425. 10.1016/j.visres.2005.04.006 15893785

[CR175] Voßkühler, A., Nordmeier, V., Kuchinke, L., & Jacobs, A. M. (2008). Ogama (open gaze and mouse analyzer): Open-source software designed to analyze eye and mouse movements in slideshow study designs. *Behavior Research Methods,* *40*, 1150–1162. 10.3758/BRM.40.4.1150 19001407

[CR176] Wartenberg, C., & Holmqvist, K. (2005). Daily newspaper layout-designers’ predictions of readers’ visual behaviour-a case study. *Lund University Cognitive Studies,* *126*, 1101–8453.

[CR177] Wenzlaff, F., Briken, P., & Dekker, A. (2018). If there’s a penis, it’s most likely a man: Investigating the social construction of gender using eye tracking. *PloS ONE,* *13*(3), e0193616. 10.1371/journal.pone.0193616 PMC5832313 29494689

[CR178] Wertheim, A. H. (2010). Visual conspicuity: A new simple standard, its reliability, validity and applicability. *Ergonomics,* *53*(3), 421–442. 10.1080/00140130903483705 20191416

[CR179] Westum, E. (2024). https://www.boredpanda.com/find-the-panda-illustrated-puzzles-openlist/. Accessed 12 Nov 2024.

[CR180] Whitney, D., & Levi, D. M. (2011). Visual crowding: A fundamental limit on conscious perception and object recognition. *Trends in Cognitive Sciences,* *15*(4), 160–168. 10.1016/j.tics.2011.02.005 PMC3070834 21420894

[CR181] Wildenmann, U., & Schaeffel, F. (2013). Variations of pupil centration and their effects on video eye tracking. *Ophthalmic and Physiological Optics,* *33*(6), 634–641. 10.1111/opo.12086 24102513

[CR182] Wolf, J., Hess, S., Bachmann, D., Lohmeyer, Q., & Meboldt, M. (2018). Automating areas of interest analysis in mobile eye tracking experiments based on machine learning. *Journal of Eye Movement Research,* *11*(6), 6. 10.16910/jemr.11.6.6 PMC7909988 33828716

[CR183] Wyatt, H. J. (2010). The human pupil and the use of video-based eyetrackers. *Vision Research,* *50*(19), 1982–1988. 10.1016/j.visres.2010.07.008 PMC2948855 20638401

[CR184] Xu, J., Jiang, M., Wang, S., Kankanhalli, M. S., & Zhao, Q. (2014). Predicting human gaze beyond pixels. *Journal of Vision,* *14*(1), 28. 10.1167/14.1.28 24474825

[CR185] Zelinsky, G., Zhang, W., Yu, B., Chen, X., & Samaras, D. (2005). The role of top-down and bottom-up processes in guiding eye movements during visual search. *Advances in Neural Information Processing Systems, 18*.

[CR186] Zhang, X., Yuan, S.-M., Chen, M.-D., & Liu, X. (2018). A complete system for analysis of video lecture based on eye tracking. *IEEE Access,* *6*, 49056–49066.

[CR187] Zhou, S., Gao, Y., Zhang, Z., Zhang, W., Meng, H., & Zhang, T. (2022). Visual behaviour and cognitive preferences of users for constituent elements in forest landscape spaces. *Forests,* *13*(1), 47.

